# Acoustic Tweezing Cytometry Induces Rapid Initiation of Human Embryonic Stem Cell Differentiation

**DOI:** 10.1038/s41598-018-30939-z

**Published:** 2018-08-28

**Authors:** Tuğba Topal, Xiaowei Hong, Xufeng Xue, Zhenzhen Fan, Ninad Kanetkar, Joe T. Nguyen, Jianping Fu, Cheri X. Deng, Paul H. Krebsbach

**Affiliations:** 1 Department of Biomedical Engineering, University of Michigan, Ann Arbor, MI 48109 USA; 2 Department of Biologic and Materials Sciences, University of Michigan, Ann Arbor, MI 48109 USA; 3 Biointerfaces Institute, University of Michigan, Ann Arbor, MI 48109 USA; 4 Department of Mechanical Engineering, University of Michigan, Ann Arbor, MI 48109 USA; 5 Department of Biomedical Engineering, Tianjin University, Tianjin, P.R. China; 6 Department of Cell and Developmental Biology, University of Michigan Medical School, Ann Arbor, MI 48109 USA; 7 Section of Periodontics, University of California, Los Angeles School of Dentistry, Los Angeles, CA 90095 USA

## Abstract

Mechanical forces play critical roles in influencing human embryonic stem cell (hESC) fate. However, it remains largely uncharacterized how local mechanical forces influence hESC behavior *in vitro*. Here, we used an ultrasound (US) technique, acoustic tweezing cytometry (ATC), to apply targeted cyclic subcellular forces to hESCs via integrin-bound microbubbles (MBs). We found that ATC-mediated cyclic forces applied for 30 min to hESCs near the edge of a colony induced immediate global responses throughout the colony, suggesting the importance of cell-cell connection in the mechanoresponsiveness of hESCs to ATC-applied forces. ATC application generated increased contractile force, enhanced calcium activity, as well as decreased expression of pluripotency transcription factors Oct4 and Nanog, leading to rapid initiation of hESC differentiation and characteristic epithelial-mesenchymal transition (EMT) events that depend on focal adhesion kinase (FAK) activation and cytoskeleton (CSK) tension. These results reveal a unique, rapid mechanoresponsiveness and community behavior of hESCs to integrin-targeted cyclic forces.

## Introduction

Human embryonic stem cells (hESCs) derived from blastocyst stage embryos can be maintained in an undifferentiated state *in vitro*^1^. A better understanding of the factors that control hESC differentiation may provide insight to the processes of human development and facilitate future clinical applications for hESCs including disease modeling^2^, drug screening^3^, and regenerative medicine^4^.

While the significance of soluble factors has been well recognized, the mechanobiology of hESCs has only recently gained interest to reveal the significant roles of the insoluble solid-state signals present in the cell microenvironment. These microenvironmental cues include variations in the rigidity and composition of the extracellular matrix (ECM), and external mechanical forces that regulate cellular behavior in human development and diseases^5–8^. In most *in vitro* studies of mechanoresponsiveness of hESCs, mechanical factors such as matrix rigidity, fluid shear stress, and stretching forces are globally applied and generally presented as a uniform and relatively stable long-term condition for all cells in a monolayer^9,10^. Under such conditions, cytoskeleton (CSK) tension and cell-ECM interactions reach a balanced state, and it is difficult to disentangle the integrated roles of external mechanical stimuli and cell-cell interaction in mechanoresponses of hESCs. Uniform culture conditions also hinder the investigation of intrinsic mechanosensitivity of hESCs to local and acute mechanical signals. Since embryonic development occurs through a dynamic process of spatiotemporally changing physical environments, it is possible that local and dynamic mechanical signals provide potent signaling cues capable of initiating important processes in hESCs such as differentiation and epithelial-mesenchymal transition (EMT), a developmental milestone by which the pluripotent epiblast commits towards differentiated paths through extensive morphogenetic changes^11,12^.

Here we employed a recently developed ultrasound (US)-based technique, acoustic tweezing cytometry (ATC)^13–15^ to investigate how hESCs respond to spatiotemporal, non-uniform mechanical forces. Since ATC only exerts cyclic forces to a cell via one or several integrin-bound MBs on cell surface, the technique provided a unique opportunity to investigate mechanoresponses of the hESCs that were directly subjected to the integrin-targeted forces and other cells in a colony that were not.

## Results

### ATC-mediated cyclic strains to integrin receptors increased cellular contractility of hESCs

**Figure 1:**
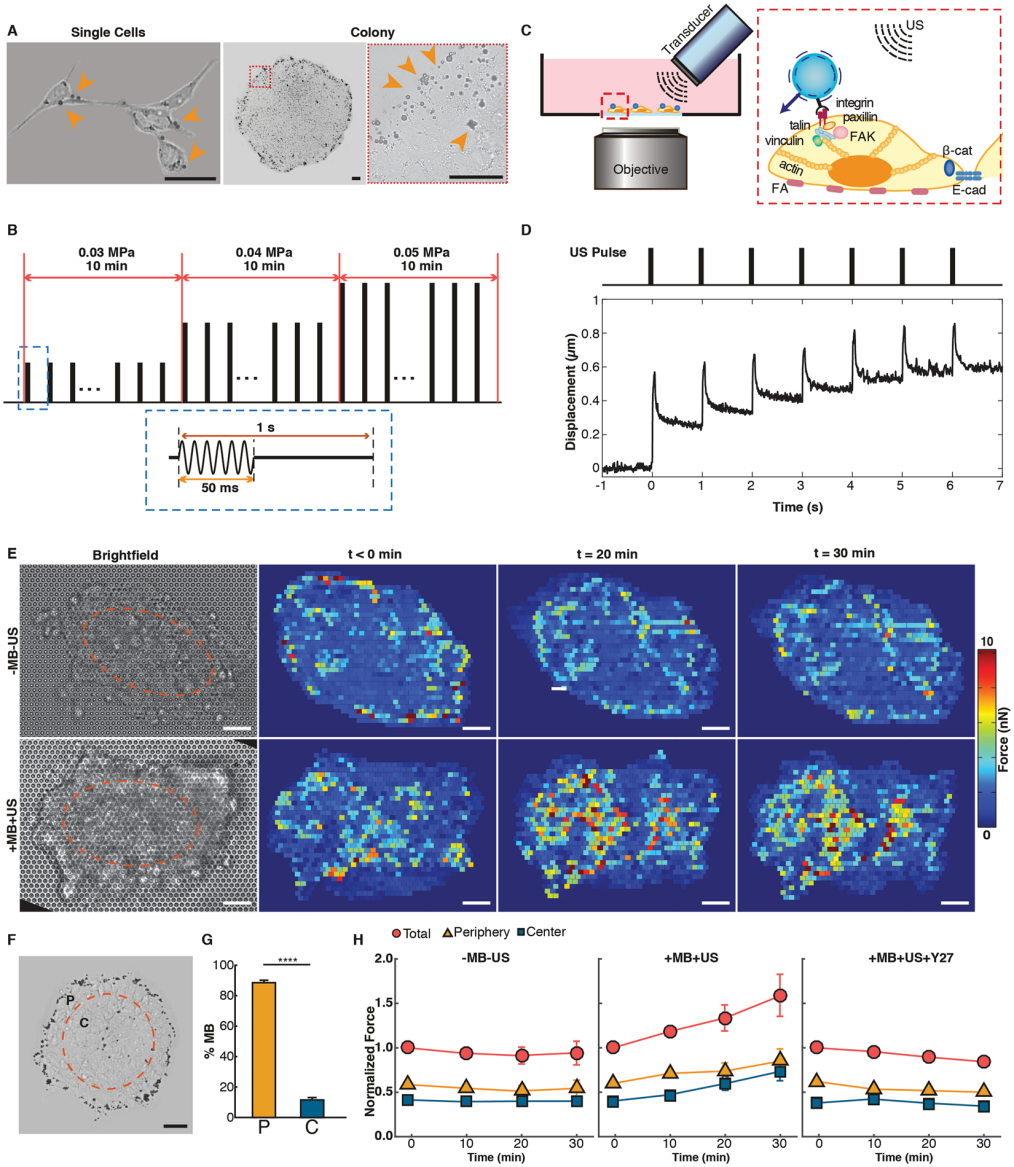
ATC application using ultrasound (US) excitation of integrin-anchored microbubbles (MBs) and increased cellular contractile forces of hESCs. (**A**) Bright field images of single hESCs and colony with integrin-bound MBs (arrow heads). Scale bar 40 µm. (**B**) US pulses used in ATC. (**C**) Experimental setup for ATC. (**D**) Cyclic MB displacement vs. time subjected to pulsed US application during ATC application. (**E**) Bright field images of hESCs seeded on PDMS micropost force sensor array and subcellular contractile force distributions in control hESC colony (-MB-US) (top) and hESC colony treated by ATC (+MB +US) (bottom) at t = 0, 20 and 30 min. ATC was applied at t = 0 for 30 min. ‘P’ denotes periphery zone and ‘C’ central zone. Scale bar 20 µm. (**F**) Bright field image showing distribution of MBs attached to hESCs in a colony. Colony area is divided into two equal regions marked by the orange circle: periphery (P) (outer 30% radius) and central zone (**C**) (center 70% radius). (**G**) Percentage of MBs attached to hESCs in colonies (*n* = 7) in the periphery (P, 88.53% ± 1.63%) and in the center (C, 11.47% ± 1.63%) (Mean ± SEM). **** denotes *p* < 0.0001. (**H**) Normalized contractile force in the entire colony, in the periphery zone, and in the center zone *vs*. time for control (-MB-US), ATC-treated (+MB +US), and Y27632 (Y27) (10 µM) treated hESC colonies (Mean ± SEM, *n* = 10).

To use ATC to apply cyclic forces to hESCs via integrins, we attached lipid-shelled microbubbles (MBs) coated with RGD peptides targeting α_5_β_3_ integrin receptors (Visistar^TM^-Integrin MBs, Targeson) (radius 2.11 ± 0.07 μm, *n* = 119) to adherent hESCs (Fig. 1A). Application of US pulses (Fig. 1B,C) exerted directional forces (17.0 ~ 25.0 nN) on the integrin-anchored MBs^15^, displacing them from their original equilibrium positions (Fig. 1D, Movies S1 and S2). The time-dependent displacement and retraction curves of the integrin-bound MBs after each US pulse reveal the characteristic viscoelastic behaviors reflecting properties of the MB-integrin-CSK linkage (Fig. 1D). Importantly, displacements of the integrin-bound MBs away from their pre-US location generated strains to the molecular RGD-integrin-CSK linkage, likely leading to stretching/distortion of the molecular assembly. Such stretching/distortion could lead to functional modification of adhesion and cytoskeletal proteins, key factors in conversion of mechanical forces into biological signals^16,17^. Interestingly, after each US pulse, these integrin-anchored MBs often retracted only partially from the peak displacement (Figs 1D and S1A–C), resulting in a gradual shift in the “resting” position of MBs (~ 0.6 µm after 7 pulses, Figs 1D and S1D). Partial retraction of MB displacements suggested partial recovery of the stretching/distortion of the adhesion and CSK proteins after the force was removed, while the shift in the MB resting location represented more permanent changes in the adhesion and/or CSK proteins due to application of US pulses.

Because cell contractility is an important morphogenetic trait responsive to external forces^18^, we measured cellular contractile forces of hESCs during ATC application using poly-(dimethylsiloxane) (PDMS) micropost arrays as real-time force sensors^13^. To examine the direct effect of cyclic force/strain to the cells and exclude the contribution of cell-cell contacts, we conducted experiments using single adherent hESCs, which were viable and maintained pluripotency 24 h after plating before our experiments. For single hESCs, cellular contractility increased immediately after ATC application in the cells with attached MBs (Fig. S2A,B), suggesting robust cellular responses to integrin-targeted external forces/strains. The single hESCs without attached MBs did not show any changes in cellular contractility even with the application of US pulses, underscoring the role of acoustic actuation of integrin-anchored MB. Because ROCK signaling is crucial for the activation of cell contractility, we hypothesized that blockage of ROCK signaling would inhibit the increase in cell contractility^19^. Indeed, when cells were treated with Y27632, a ROCK pathway specific inhibitor^20,21^, increase of cell contractility was dramatically abated (Fig. S2B), suggesting that increased contractility of single hESCs subjected to ATC-mediated integrin-targeted forces requires tension in an intact CSK.

Interestingly, ATC application increased cellular contractility in hESCs throughout a colony even though some of the hESCs in the colony did not have attached MBs (Fig. 1E), and the effect was abolished by Y27632 (Fig. 1H). In these experiments, MBs were observed to be preferentially attached to the hESCs near the colony periphery (Fig. 1F,G), with 1.44 ± 0.14 MBs/cell (456 ± 80 MBs, *n* = 7) in the peripheral zone (defined as the region occupying the outer 30% of colony radius) and 0.17 ± 0.03 MBs/cell (61 ± 13 MBs, *n* = 7) in the central zone (region within the inner 70% of colony radius), respectively. Thus, many cells in the colony’s central zone did not have MBs attached to them, thus were not directly subjected to forces via integrin-bound MBs during ATC application. Despite the fact that only some cells were directly subjected to integrin-targeted strain/force, ATC application increased cellular contractility globally throughout the colony (Fig. 1E,H), highlighting the critical role of cell-cell communication in generating mechanoresponses in hESCs in a colony. Interestingly, while the cells in the central zone exhibited lower contractile forces than those in the peripheral zone before ATC application (Fig. 1E,H)^22^, they sustained greater increases in contractility as shown by the larger slope of force increase (center panel in Fig. 1H) for these cells to match the level in the periphery during ATC application (Fig. 1H), resulting in mechanical homogenization of a hESC colony. Particularly, since only a subpopulation of the hESCs in the colony were subjected directly to the ATC-mediated forces, this global response suggests an important role of cell-cell communication in the mechanoresponsiveness of hESC colony to integrin-targeted cyclic forces.

In addition to global changes in cellular contractility, we also observed that ATC application induced intracellular calcium activities throughout entire hESC colonies, even though a subpopulation of cells in the colony were subjected by direct forces via integrin by attached MBs during US application (Movie S3 and Fig. S3). Since spontaneous calcium activities do not occur in undifferentiated hESCs^23^, these results suggested another aspect of the collective mechanoresponse of hESC colony to external forces via integrin, which may indicate differentiation of these cells.

### Cyclic forces to integrins induced rapid decrease of Nanog and Oct4 in hESCs

**Figure 2:**
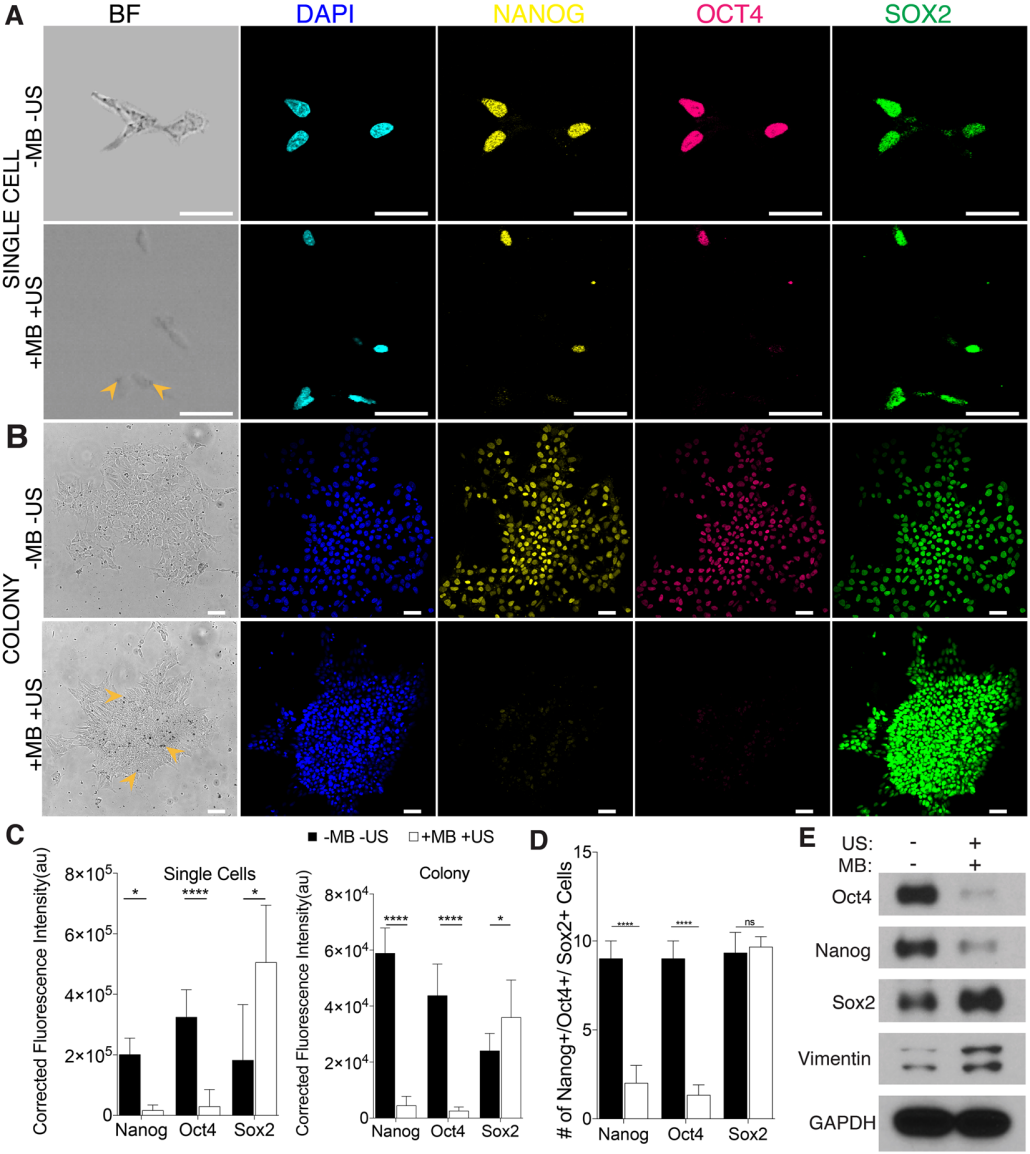
ATC application downregulated pluripotency markers in hESCs. (**A**) Bright field images of single ESCs and integrin-bound MBs (arrows). Confocal fluorescence images of single hESCs stained with DAPI (blue), Oct4 (magenta), Nanog (yellow), and Sox2 (green) with and without ATC application (30 min). Scale bars: 50 µm. (**B**) Percentages of hESCs with Oct4, Nanog, and Sox2 after ATC treatment compared with control. (**C**) Bright field images of a hESC colony and integrin-bound MBs (arrows). Confocal fluorescence images showing hESCs stained with DAPI (blue), Oct4 (magenta), Nanog (yellow), and Sox2 (green) with and without ATC application. Scale bars: 50 µm. (**D**) Corrected fluorescence intensity measurement of hESCs with Nanog, Oct4, and Sox2 after ATC treatment. (**E**) Immunoblot analysis of protein expression in hESCs. All quantifications were from at least 3 independent experiments with two replicates per experiment. Unpaired *t* test *p* values * < 0.05, **<0.01, and ***<0.001; n.s, not significant.

We next examined transcriptional response of hESCs to ATC application. For single hESCs targeted by MBs (arrow), rapid decease in Oct4 and Nanog was clearly seen after just 30 min application of US pulses, while Sox2 was not significantly affected (Fig. 2A,B). In contrast, single hESCs without attached MBs, although exposed to the same US pulses, exhibited no change in Oct4 or Nanog (arrow in Fig. 2A), suggesting that these transcription factors responded directly to integrin-targeted cyclic forces for single hESCs. Interestingly, for hESC colonies, even though MBs were mostly attached to the cells near the colony periphery (Fig. 2C), ATC application for 30 min also decreased Oct4 and Nanog in the entire colony (Fig. 2C,D), suggesting a collective mechanoresponse of hESC colony to locally applied external cyclic forces. These effects were not observed in hESC colonies without attached MBs or without US application (Fig. S4A,B,D). In addition, the ATC-induced decreases in Oct4 and Nanog were irreversible, as shown by immunostaining results after 48 hours of culture after ATC treatment (Fig. S5). Mirroring the global increases in cellular contractility and calcium activities in hESC colonies (Figs 1E and S3), these ATC-induced global transcription responses within the entire colony demonstrate the community characteristics of hESC mechanoresponses and initiation of differentiation induced by local integrin-targeted cyclic forces.

Immunoblot analysis confirmed decreased expression of Oct4 and Nanog following ATC treatment (Figs 2E, S14), while in other control groups (−MB +US and +MB −US), no change was observed (Fig. S4C). Gene expression analysis showed no change in Nanog, Oct4, and Sox2, while a decrease was observed in neuroepitelial (NE) marker, Pax6 in microbubble attached and ultrasound treated group (Fig. S4E). Comparable effects of ATC-mediated forces were observed in another hESC line (H1) (Fig. S6).

Although direct cyclic forces via integrin-anchored MBs were only applied to a fraction of the cells in the colony with attached MBs, the changes in OCT4, Nanog, and Sox2 expression were global and were observed throughout the colony (Fig. 1). These results highlight the involvement of cell-cell communication of the cells in the colony in their response the integrin-targeted cyclic forces.

Taken together, these results highlight a remarkable mechnosensitivity and community characteristics of hESCs to locally applied integrin-targeted cyclic forces/strains, which induced rapid loss of pluripotency and initiation of hESC differentiation in the entire colony immediately after 30 min of ATC treatment, an observation in distinct contrast to the multiple-day process needed for transcriptional changes in differentiation induced by soluble factors or regulated by uniform matrix rigidity^9^.

### Cyclic forces to hESCs activated focal adhesion kinase (FAK) and a switch from E- to N-cadherin

**Figure 3:**
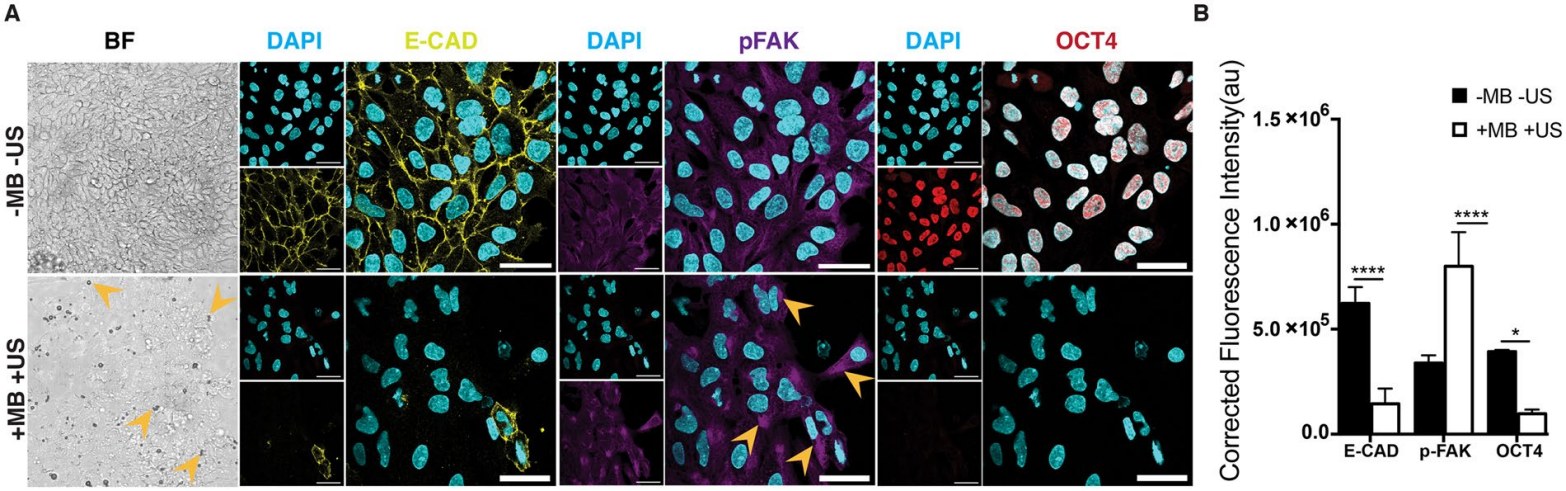
ATC application activated FAK signaling and induced differentiation of hESCs. (**A**) Adherent colony of hESCs stained with DAPI (blue), E-cadherin (yellow) and pFAK (purple) with and without ATC stimulation (30 min). (**B**) Corrected fluorescence intensity of E-cadherin, pFAK activation and Oct4 after 30 min ATC stimulation compared with control group. Scale bars 50 µm. All quantifications were from at least 3 independent experiments with two replicates per experiment. Unpaired *t* test *p* values *<0.05, **<0.01, and ***<0.001. n.s. not significant.

Since FAK is an important mechanotransductive component downstream of force-activated integrin^24^, we examined whether ATC application influenced changes in FAK activity in hESCs. We detected phosphorylated FAK (pFAK) in the cytoplasm of hESCs subjected to ATC stimulation, and the percentage of cells with cytoplasmic pFAK increased significantly compared to untreated controls (Fig. 3B). These results are consistent with a recent result of pFAK in differentiated hESCs^25^. No change was observed in the cells without attached microbubbles with ultrasound (−MB +US) and with attached microbubble and without ultrasound (+MB −US) treatment groups (Fig. S7A,B). In addition, a decrease was observed in integrin alpha V (ITGAV) and FAK (PTK2) gene expression in microbubble attached and ultrasound treated group while no change was observed in control groups (Fig S7C). In addition, we also found that loss of Oct4 and Nanog in hESCs due to ATC application was accompanied with decreased E-cadherin expression (Fig. 3A,B)^26,27^. These results support the role of E-cadherin in maintenance of hESC pluripotency^20,27,28^, further suggesting loss of pluripotency and initiation of differentiation of hESCs resulted from ATC application.

**Figure 4:**
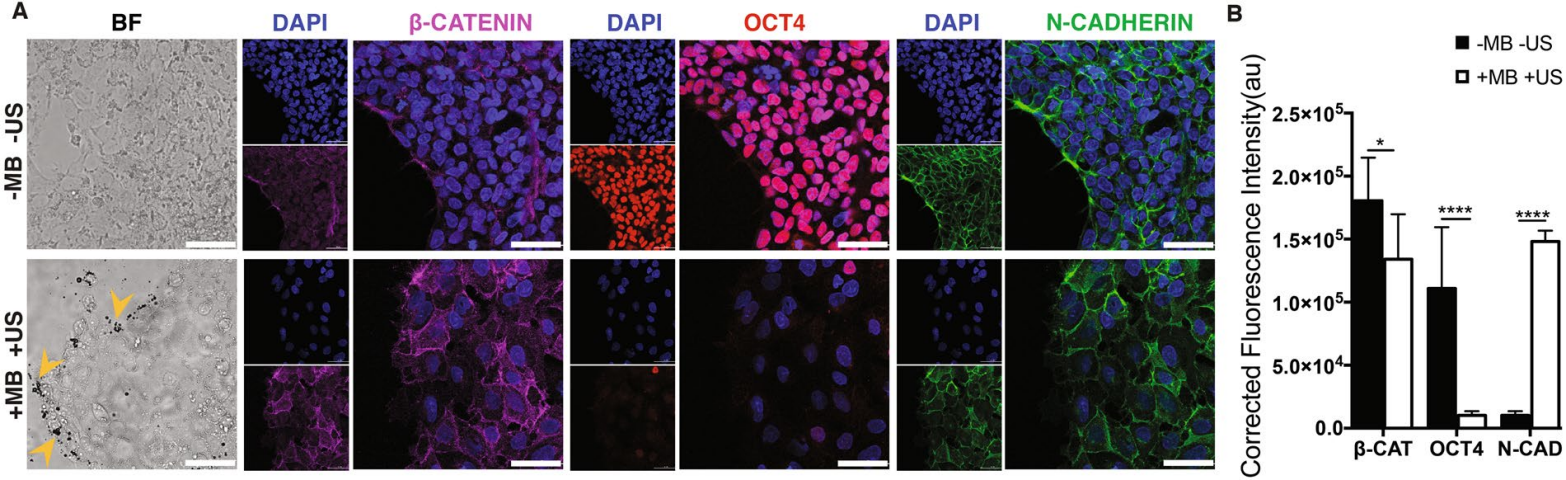
ATC application induced differentiation of hESCs by elevated levels of N-cadherin and decreased β-Catenin expresion (**A**) Adherent colony of hESCs stained with DAPI (blue), β-Catenin (yellow), and N-Cadherin (green) after 30 min of ATC stimulation vs. control. (**B**) Corrected fluorescence intensity of β-catenin, N-cadherin, and Oct4 expression in hESCs after 30 min of ATC stimulation compared control. Scale bars 50 µm. All quantifications were from at least 3 independent experiments with two replicates per experiment. Unpaired *t* test *p* values *<0.05, **<0.01, and ***<0.001. n.s. not significant.

Unlike E-cadherin, N-cadherin is not expressed in undifferentiated hESCs, and cadherin switching from E- to N-cadherin is crucial for morphogenetic movement in embryonic development^20,28^. Interestingly, ATC treatment elevated N-cadherin expression hESCs, accompanied by significantly decreased Oct4 and β-catenin expression (Fig. 4A,B). No change was observed in the group without attached microbubbles with ultrasound (−MB +US) and the group with attached microbubble and without ultrasound application (+MB −US) (Fig. S8). When hESCs were pre-treated with PF562271 (10 mM) to inhibit FAK activity, minimal changes in N-cadherin, pFAK or Oct4 expression were observed with ATC stimulation (Fig. S9A,B). Treatment with blebbistatin (100 µM) also abrogated expression changes in β-catenin, E-cadherin and Oct4 due to ATC application (Fig. S9C,D). Taken together, these results demonstrate that cyclic force-mediated loss of pluripotency and initiation of hESC differentiation in a colony required FAK activation and myosin II activity.

### Cyclic forces applied to integrins induced rapid epithelial-mesenchymal transition (EMT) in hESCs

**Figure 5:**
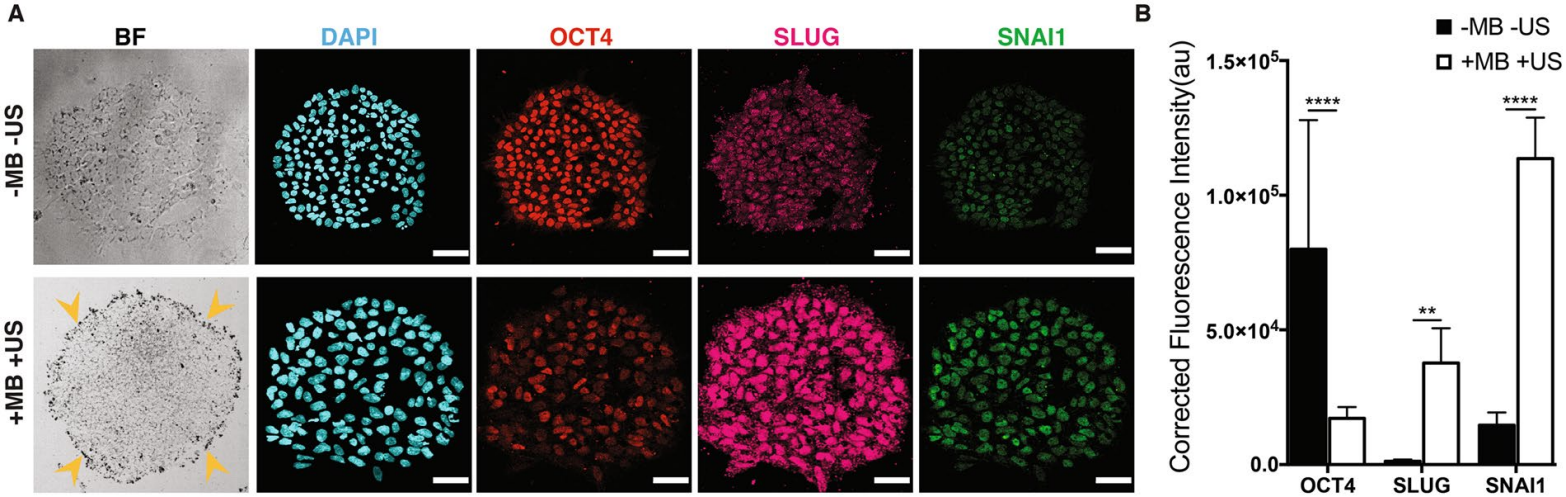
ATC application induced differentiation and EMT events in hESC colonies. (**A**) Colony of hESCs stained with DAPI (blue), Oct4 (magenta), Slug (yellow), and Snai1 (green) with and without ATC stimulation (30 min). (**B**) Corrected fluorescence intensity of Oct4, Snail, and Slug expression in the cells after 30 min of ATC stimulation compared with control. Scale bars 50 µm. All quantifications were from at least 3 independent experiments with two replicate monolayers per experiment. Unpaired *t* test *p* values *<0.05, **<0.01, and ***<0.001. n.s. not significant.

As the ATC-induced loss of pluripotency and initiation of differentiation of hESCs in monolayer culture is associated with characteristic EMT events such as an E- to N-cadherin switch (Fig. 3), we examined the extent of ATC induced up-regulation of E-cadherin repressor molecules in hESC colonies^27,28^. Indeed, ATC application significantly upregulated the transcription factors Snail and Slug, while decreasing Oct4 in hESCs (Fig. 5A,B). No change was observed in the group without attached microbubbles with ultrasound (−MB +US) and the group with attached microbubble and without ultrasound (+MB −US) (Fig. S10). In addition, hESCs subjected to ATC treatment exhibited a significant increase in T (brachyury) expression (Fig. S11A,B). No change was observed in the group without attached microbubbles with ultrasound (−MB +US) and the group with attached microbubble and without ultrasound (+MB −US) (Fig. S10C,D). No change was observed in T and Snai1 gene expression (Fig. S11C). These results demonstrated that 30 min application of ATC-mediated cyclic forces/strains to integrin induced rapid initiation of differentiation and EMT in hESC colonies, in stark contrast with the multiple-day process of differentiation and EMT in hESCs in the presence of morphogenic factors or regulated by matrix rigidity^27–29^.

### ATC-mediated cyclic forces inhibit YAP nuclear localization in hESCs

Yes-associated protein (YAP) is critically involved in mechanotransduction^30^. We conducted experiments to examine YAP activity due to ATC application. We found that 30 min ATC treatment induced translocation of YAP from the nucleus to the cytoplasm of hESCs (Fig. S12A,B), and treatment with blebbistatin abolished this effect (Fig. S12C,D), suggesting a possible role of YAP and CSK tension in ATC-induced hESC phenotypic change. These results are consistent with the notion that force-mediated Hippo/YAP activities and CSK contractility are involved in mechanotransductive processes in hESCs^9^. It has been shown that stiff surfaces promote nuclear localization of YAP to facilitate hESC expansion^31^, and compliant substrates inhibit nuclear localization of YAP, promoting efficient differentiation of hPSCs into postmitotic neurons^9,32^. However, ATC-mediated cyclic forces increased cell contractility while promoting cytoplasmic YAP localization in differentiating hESCs. These results suggests that ATC induced initiation of hESC differentiation in a different manner than substrate rigidity, highlighting the unique mode of integrin-targeted cyclic forces in directing hESC fate.

## Discussion

It is well known in other organisms that the embryo experiences tension and compression forces before and after gastrulation, i.e. attachment to the uterine wall and beginning the development processes, with many cells enacting their forces on the embryo to facilitate development. However, as the magnitude and duration of the actual forces experienced by earlier embryos encountered during implantation is difficult to obtain, it is difficult to make direct comparison between the ATC induced forces and those forces experienced in the *in vivo* environment. Nevertheless, ATC application exerted cyclic forces with a repetition frequency and amplitude that can be readily adjusted. In this work, we chose 1 Hz for the cyclic force application and about 15 nN for the magnitude, which is generally in the same order of that of typical physiological biomechanical rhythms. The short duration of ATC application in our study makes it likely to represent an acute, dynamic mechanical cue that initiated subsequent changes.

Our results demonstrate that direct cyclic force and strain applied to integrin receptors of hESCs for 30 min generated rapid mechanoresponses in single hESCs and hESC colonies, inducing enhancement of cellular contractility, calcium activity, decrease in expression of Oct4 and Nanog, as well as events associated with EMT. For single cells and colonies, the cyclic forces not only induced changes in the cells with attached MBs, but also the cells throughout the colony. As not all the cells in the colony had attached MBs, our results reveal the orchestration of mechanoresponses in hESC colony through integrin and cell-to-cell contacts. As a result, hESCs in a colony subjected to locally applied cyclic forces/strains via integrin by ATC application progress as a homogeneous population from a pluripotent state with downregulation of transcription factors of Oct4 and Nanog, exhibiting EMT with a loss of E-cadherin and gain of migratory properties by N-cadherin expression, which were accompanied by Snail, Slug, as well as Ki-67 expression. It is possible that such responses to ATC induced cyclic forces are amplitude- and duration dependent. Whether a threshold of ATC application exists for these phenomena remains to be examined.

In this study, we demonstrate that ATC application, by displacing integrin-bound microbubbles to hESCs, applied targeted forces to generate cyclic strains to individual cells with a subcellular resolution. Since US pulses used in ATC can be broadly applied to actuate integrin-bound MBs, our technique provides a high throughput strategy that can be used to study a large number of hESCs *in situ* simultaneously. This provides a distinct advantage over other established biophysical techniques such as optical tweezer and atomic force microscopy (AFM) which are limited to single cell analysis. Furthermore, our results show that this technique does not lead cell death based on our cell viability assay results (Fig. S13A–G). Our results demonstrate the potential of ATC as a new biomechanical technique that could be used for mechanobiology studies and for potentially improving the differentiation efficiency of existing protocols using growth factors for differentiation of hESCs.

Beyond this feasibility study, further work is needed to explore the dose-effect relationship of magnitude and duration of mechanical forces applied to integrin of hESCs by ATC and the ensuing effects. Additionally, the definitive signaling pathways and molecular players that are involved in the rapid initiation of hESC differentiation in a colony induced by locally applied cyclic forces to integrin, and the integration of cell-cell contacts in the mechanoresponses of hESCs, will need to be elucidated. Future studies are needed to understand the end point of differentiation including the specific germ-layers (endo-meso-ectoderm) derived from these differentiating cells initiated by ATC application in order to shed light on how mechanical cues are involved in control hESC fate for the benefit of developmental biology and regenerative therapies.

## Materials and Methods

### Matrigel preparation

Matrigel (Corning® Matrigel® hESC-Qualified Matrix, *LDEV-Free, cat# #354277) was diluted to a concentration of 0.1 mg/ml in cold Dulbecco’s modified Eagle’s medium/F12 (DMEM/F12; GIBCO) and then applied to glass bottom tissue culture polystyrene (TCPS) dishes (35 mm; 10 mm glass diameter, MatTek Corporation cat# P35G- 1.5-10-C). The coating was allowed to polymerize for 2 hours incubation at room temperature. Excess Matrigel-DMEM/F12 solution was aspirated before plating cells, and then dishes were washed with sterilized Dulbecco’s phosphate buffered saline (D-PBS).

### hESC culture

hESCs (H9 and H1, WiCell Research Institute, Madison, WI) were cultured on the synthetic surface PMEDSAH as described previously^33^ with human-cell- conditioned medium (HCCM, MTI-Global Stem, Gaithersburg, MD, http://www.mti-globalstem.com) supplemented with 5 ng/mL of human recombinant basic fibroblast growth factor (FGF2; InvitrogenTM, Carlsbad, CA, http://www.invitrogen.com), and 1%antibiotic-antimycotic (Gibco). The hPSC culture medium was replaced every other day and all cell culture was performed in designated incubators at 37 °C in 5% CO_2_ and high humidity. Differentiated cells were mechanically removed using a sterile pulled-glass pipet under a stereomicroscope (LeicaMZ9.5, Leica Microsystems Inc., Buffalo Grove, IL).

#### For single cells

Undifferentiated colonies were washed with PBS and a non-enzymatic cell attachment passaging solution (Lonza L7 hPSC) was added for gentle disassociation of colonies and incubated at 37 °C for 5 min. L7 was removed and 2 ml of HCCM was added, and then cells were scraped and collected into conical tube for brief centrifugation. The cell pellet was dispersed in HCCM supplemented with 5 ng/mL bFGF and 10 mM of ROCK inhibitor (Sigma) (26) and passed through a 40 mm nylon mesh cell strainer (BD Biosciences, Bedford, MA) to remove large cell aggregates. Single hESCs were counted and 100 K/ml cells were plated on Matrigel coated glass dishes cultured for overnight for attachment

#### For colonies

Undifferentiated colonies were cut into small pieces and transferred them onto matrigel coated glass dishes (~5–6 as small cell clusters for each plate), and let them to attach for overnight.

(Unless otherwise specified, hESCs (H1, NIH registration # 0043and H9, NIH registration number # 0062 from WiCell ResearchInstitute, Madison, WI, http://www.wicell.org.

### Attachment of targeted lipid microbubble to cells

Visistar^TM^-Integrin lipid microbubbles (MBs) with high affinity for α5β3 integrin (mean ± SE of bubble radius: 2.11 ± 0.07 μm, n = 119; Targeson) were diluted to a final concentration of 2 * 10^7^ MBs mL^−1^ in HCCM. Cell culture in Matrigel-coated petri dish was gently washed with D-PBS, and loaded with 100 μL diluted MBs in culture media. The petri dish was flipped upside down and incubated at 37 °C for 10 min to enable attachment of MBs to hESCs via RGD-integrin binding, followed by another gentle wash with culture media to remove unbound MBs.

### Ultrasound stimulation

A planar ultrasound (US) transducer with 1 MHz center frequency (−6 dB beam width of 3.54 mm at a Rayleigh distance of 9 mm; Advanced Devices) was used for ATC application. The US transducer was positioned at an angle of 45° relative to the horizontal direction facing downward, and at 9 mm from the cells seeded at the bottom of the petri dish. The petri dish was filled with culture media to submerge the active surface of the transducer and allow acoustic coupling. The US transducer was driven by two waveform generators (Agilent Technologies) and a 75 W power amplifier (Amplifier Research) to generate cyclic pulses with 1 Hz pulse repetition frequency (PRF) and 50% duty cycle. The US transducer was calibrated in free field in water using a needle hydrophone (HNR-0500, Onda). The cells were exposed to 30 min ultrasound treatment at an acoustic pressure level ramping from 0.03 to 0.04 and 0.05 MPa, 10 min at each level.

The primary acoustic radiation force was estimated as:

$${\rm{F}}=2{{{\rm{\pi }}{\rm{P}}}_{{\rm{A}}}}^{{\rm{2}}}{{\rm{DR}}}_{{\rm{0}}}/{{\rm{\delta }}}_{{\rm{tot}}}{{\rm{\rho }}}_{0}{{\rm{c}}{\rm{\omega }}}_{0}{\rm{T}}$$

where *P*_*A*_ is the acoustic pressure, D is the pulse duration (50 ms), *R*_0_ is the bubble radius, *δ*_*tot*_ is the total damping constant (*δ*_*tot*_ = 0.16), *ρ*_0_ is the medium density (1000 kg m^−3^), and *c* is the sound speed in media (1500 m s^−1^), *ω*_0_ is the resonance frequency (1.25 MHz), and *T* is the pulse repetition period (1 s)^34^.

To observe the MB dynamics, images of the MBs exposed to ultrasound stimulation were captured with a high-speed camera (FASTCAM SA1, Photron) at a frame rate of 2000 frame/s. MB radius and location in each frame were quantified and tracked over time using an automated MATLAB script.

### Contractile force quantification using PDMS micropost array

Contractile force measurements were performed as described before^35^. Briefly, hESCs were seeded on PDMS micropost array (PMA) either as single cells or colonies, and incubated overnight to allow attachment. The PMA top surface was coated with fibronectin (Sigma-Aldrich) to promote cell adhesion. Microposts with 1.83 μm diameter, 7.1 μm height, and a spring constant of 7.22 nM/μm were used in this study. Fluorescence images of the PMA top surface were captured throughout the ultrasound treatment using an inverted epi-fluorescence microscope enclosed in a cage incubator (Carl Zeiss MicroImaging) maintaining the temperature at 37 °C and the CO_2_ at 5%. Using a custom-written MATLAB script, micropost deflections were detected, quantified, and multiplied by the micropost spring constant to produce the horizontal contractile force.

#### Inhibition experiments

PF-562271 (Sigma-Aldrich) was added to the culture media at 1 µM an hour before ultrasound treatment to inhibit focal adhesion kinase phosphorylation. To inhibit the Rock pathway and the myosin-II heavy chain, Y27632 and blebbistatin (Sigma-Aldrich) was added to the media respectively at 10 µM 30 min before applying ultrasound stimulation.

### Statistical Analysis

Results are presented as Mean ± SEM. Unpaired two-tailed student’s t test was performed for comparisons, and a p-value < 0.05 was considered statistically significant.

### Immunoblot analysis

Cells were collected and lysed in NP40 lysis buffer (NaCl at 150 mM, NP-40 at 1.0%, and TrisCl at 50 mM, pH 8.0). Supernatant was quantified using Bio-Rad Protein Assay (Bio-Rad; cat# 5000006) with the Biotek Nova Spectrophotometer at 595 nm wavelength. 10 μg of protein lysate was mixed with 3x Laemmli loading buffer (4% SDS, 10% 2-mercaptoethanol, 20% glycerol, 0.004% bromophenol blue, 0.125 M Tris-HCl, brought to pH 6.8). Novex® Tris-Glycine SDS Running Buffer 10X(TFS: cat# LC2675-4) was diluted to 1X with milliQ filtered deionized water. We used Novex™ WedgeWell™ 4–20% Tris-Glycine Gels (TFS; cat# XP04205BOX) and XCell SureLock® Mini-Cell (TFS; cat# EI0001). For optimal separation of proteins ranging 50 to 75 kDa for S6K2 phosphorylation immunoprecipitation analysis, we used the Novex™ WedgeWell™ 10% Tris-Glycine Gels (TFS; cat# XP00105BOX). Precision Plus Protein™ Dual Color Standards (Bio-Rad; cat# 1610374) to approximate molecular weights from 10 kDa to 250 kDa. Gels are run at 150 V for 85 min. For transfer of proteins, we use Trans-Blot® SD Semi-Dry Transfer Cell (Bio-Rad; cat# 170-3940) and PVDF membranes dipped in methanol briefly and rinsed in 1X Transfer Buffer (25 mM Tris, 192 mM glycine, 20% methanol (v/v), 0.5% SDS at pH 8.3) (Bio-Rad; cat# 162-0177 R) to transfer 24 V for 60 min. After the transfer, membranes were blocked for 30 min in a 2.5% blocking solution (Bio-Rad; cat# 1706404) in 50 mL 1X TBST (50 mM Tris-Cl, pH 7.5, 150 mM NaCl, Tween-20 (FS; cat# BP337-100) for 30 min, followed by 1x wash for 30 min in 1X TBST. Prior to antibody incubation, membranes were cut between the 75 kDa and 100 kDa bands to optimize for proteins above 100 kDa in size, above 75 kDa and above the 37 kDa membrane for proteins within those ranges, and between 20–25 kDa for proteins above and below those ranges. This allows for optimal antibody incubation with the proper membranes. After incubating membranes in primary antibodies overnight at 4 °C, they were washed for 30 min in 1X TBST at room temperature (RT), shaking. Next, membranes were incubated in the appropriate secondary antibody for one hour and washed for 30 min in 1X TBST at RT. For film capture, membranes were incubated with SuperSignal™ West Pico Chemiluminescent Substrate (TFS; cat# 34078) for 3 min at RT. We used HyBlot CL autoradiography film (Denville Scientific: cat# e3018). Primary antibodies for immunoblot analysis: E-cadherin, p-FAK (Tyr^397^), p-YAP (S127), Ki-67, Vimentin, and Oct4 were used at 1:1,000 dilution in 5% BSA in 1X TBST buffer with 0.04% sodium azide. Almost all antibodies from Cell Signaling Technologies (CST) are rabbit: Oct4 (Cell Signaling Technologies (CST): cat# #2750 S), p-FAK(Y397) (CST: cat#13008 S), Vimentin (D21H3)(CST: cat# 57419), p-YAP(S127) (CST: cat# 13008 S), E-cadherin (CST:mouse, cat#14472), Ki-67 (CST: mouse cat #9449). Secondary antibodies for immunoblot analysis: 1:4,000 dilution for α-mouse IgG (H + L) HRP conjugate (Promega; cat# W4021). 1:7,500 dilution for α-rabbit IgG (H + L) HRP conjugate (Promega; cat# W4011).

### Cell immunofluorescence analysis

hESCs on MatTek Corporation glass bottom dishes were washed with PBS for 5 min and aspirated out the supernant and addded 1 ml of Z-Fix solution (Anatech LTD: cat# 170) for 10 min at RT shaking. Next, we washed 3x with 1 mL of PBS for 10 min at RT. Next, we added unmasking solution (PBS, 2N HCL, 0.5% TritonX) for 10 min, removed it, added quenching solution (TBS, 0.1% Sodium Borohydride) for 10 min, removed it, added permeabilization solution (PBS, 0.02% TritonX) for 10 min. Then, we blocked with 1 mL of 5% BSA in 1X PBS for 30 min. Next, glass bottom dishes were incubated in primary antibodies overnight at 4 °C shaking. Slides were then washed with 1X PBS 3 times for 10 min at RT while shaking. We then incubated in secondary antibodies covered in foil for at least one hour at RT while shaking. Afterwards, we washed twice in PBS for 10 min, incubated in DAPI solution for 10 min and washed in 1X PBS for 10 min. Finally, we added 1 drop of ProLong® Gold Antifade Mountant onto each well, placed a glass cover slip, and sealed with acrylic nail polish. We used Nikon Ti Eclipse Confocal Microscope, 20x and 60x magnification lenses, with water to capture images. We captured images with or without 3x digital zoom, ¼ frames per second, 512 × 512 image capture, 1.2 Airy Units, 2x line averaging, appropriate voltage and power settings optimized per antibody. No modification was done, except image sizing reduction, rotation, or gray scale change for figure preparation. All original and unaltered blots are found in the supplementary figures. Primary antibodies for immunofluorescence: All antibodies were used as following with a working volume of 1 mL in 5% BSA in PBS, unless noted otherwise:

Oct4 (SantaCruz: goat, 1:500, cat# sc8629), Nanog (Abcam: mouse, 1:100 cat#ab62734), Sox2 (Millipore: rabbit, 1:500, cat# ab5603), E-cadherin (R&D Systems: mouse, 1:100, cat# AF648), pFAK (Invitrogen: Tyr 397, 31H5L17; rabbit, 1:500, cat# 700255), b-catenin (CST: rabbit, 1:100, cat#8480), N-cadherin (CST: mouse, 1:100, cat# 13116), YAP (SantaCruz: mouse, 1:500, cat#sc101199), Snai1 (SantaCruz: mouse, 1:500, cat# sc271977), Slug (CST: rabbit, 1:400, cat# 9585), and T(brachyury) (SantaCruz: rabbit, 1:500, cat# sc20109). Secondary antibodies for immunofluorescence: All antibodies were used at a concentration of 1:1,500 with a working volume of 1.5 mL in 5% BSA in PBS. DAPI stain was used for DNA. Donkey anti-Mouse IgG Secondary Antibody, Alexa Fluor® 488 (TFS: cat# R37114). Donkey anti-Rabbit IgG Secondary Antibody, Alexa Fluor® 594 (TFS: cat# A-21207).

### Image Analysis

ImageJ was used to quantify fluorescent intensity^36,37^. Briefly, after selecting the cell of interest using any of the polygon drawing/selection tools, “set measurement” was selected from the Analyze menu by selecting area, integrated density and mean gray value. Then, “Measure” was selected from the analyze menu, and a region next to the cell that has no fluorescence was selected for background. This step was repeated for each single cell in the field and then the all data were analyzed on excel, and this formula was followed for the corrected total cell fluorescence (CTCF).

$$\begin{array}{c}{\rm{CTCF}}={\rm{Integrated}}\,{\rm{Density}}-({\rm{Area}}\,{\rm{of}}\,{\rm{selected}}\,{\rm{cell}}\\ \,\,\,\,\,\,\times \,{\rm{Mean}}\,{\rm{fluorescence}}\,{\rm{of}}\,{\rm{background}}\,{\rm{readings}})\end{array}$$

### Extraction and purification of total RNA

Plates were washed with PBS and 1000 µl of Trizol Reagent (Invitrogen, Carlsbad, CA) was added to the plates, and RNAs were collected after vigorous pipetting. 200 µl Chloroform was added to this solution followed by centrifugation (13,000 g-15 min). Aqueous phase containing RNA was separated and 500 µl isopropanol was added and stored at 20 °C at least overnight. Then, the manufacturer’s RNA Clean-up protocol, RNeasy Mini-Kit (Qiagen, Valencia, CA), with the optional On-column DNAse treatment was followed. RNA quality and concentration were checked using a Synergy NEO HTS Multi-Mode Microplate Reader (BioTek Instruments, Winooski, VT).

### Reverse-transcription PCR (RT-PCR) analysis

Reverse transcription from 2.5 µg of total RNA in a 20 µL reaction into cDNA was performed using SuperScript™ VILO™ Master Mix (ThermoFisher Cat#11755050). The synthesis of first-stranded cDNA was carried out in the PCR tube after combining SuperScript VILO, RNA, and DEPC-treated water, in the first cycle at 25 °C for 10 min, incubating at 42 °C for 60 min, and terminating the reaction at 85 °C for 5 min. Quantitative PCR was performed triplicate for for each sample using TaqMan probes (Applied Biosystems) and TaqMan Universal PCR Master Mix (Applied Biosystems) on 7900 HT Fast Real Time PCR system (Applied Biosystems). Relative quantification of Nanog, Oct4, Sox2, FAK, Snai1, T, ITGAV, and Pax6 gene expression data were normalized to the GAPDH expression and calculated using the 2^−ΔΔCT^ expression level^38^.

List of primers used in qRT-PCR in Table 1. All primers were purchased from ThermoFisher Life Technologies.

**Table 1:** List of Primers.

Gene Symbol	Assay ID	UniGene ID
NANOG	Hs02387400_g1	Hs.635882
POU5F1 (OCT 3/4)	Hs03005111_g1 (FAM-MGB)	Hs.249184
SOX2	Hs01053049 (FAM-MGB)	Hs.518438
FAK (PTK2)	Hs03657683	Hs. 395482
SNAI1	Hs00195591_m1	Hs. 48029
T (BRACHURY)	Hs00610080_m1	Hs. 389457
ITGAV	Hs00233808_m1	Hs. 436873
PAX6	Hs00240871_m1	Hs.446336
GAPDH	Hs02786624_g1	Hs.544577

### Fluorescence Ratiometric calcium imaging

The cells were loaded with Fura2-AM by incubating in the culture medium dissolved with 10 µM Fura2-AM (Invitrogen) and 0.05%vol/vol of 10% wt/vol Pluronic F-127 (Invitrogen) for 60 minutes. After incubation, excess dye was removed by washing three times. Fast speed fluorescence imaging was performed using a cooled CCD camera (Photometrics QuantEM), a monochromator with 5-nm bandpass (DeltaRAM X^TM^, PTI), and a polychroic filter (73000v2, Chroma). The samples were excited at 340 nm and 380 nm alternatively, and emission fluorescence was collected at 520 nm. The pseudocolored ratio images of [Ca^2+^]_i_ based on two excitation channels were generated using Matlab (Mathworks).

## Electronic supplementary material

Movie 1

Movie 2

Movie 3

Supplementary Data Set 1

## Data Availability

All data from this manuscript is available upon request.
