# Supplementary material for: Acoustic Tweezing Cytometry Induces Rapid Initiation of Human Embryonic Stem Cell Differentiation
Source: Sci Rep. 2018 Aug 28;8:12977. doi: 10.1038/s41598-018-30939-z (PMC6113316; doi:10.1038/s41598-018-30939-z)
Supplement: Supplementary file 4 — Supplementary Data Set 1 [file 41598_2018_30939_MOESM4_ESM.docx]

**Supplementary Materials**

Acoustic Tweezing Cytometry Induces Rapid Differentiation of Human Embryonic Stem Cells

Tuğba Topal,^1,2,3§^ Xiaowei Hong,^1§^ Xufeng Xue,^4^  Zhenzhen Fan,^1,5^ Ninad Kanetkar,^1^ Joe T. Nguyen,^2,3^ Jianping Fu,^1,4,6^  Cheri. X. Deng,^1,4*^ and Paul H. Krebsbach^1,2,3,7*^

**Affiliations**

^1^Department of Biomedical Engineering, University of Michigan, Ann Arbor, MI 48109, USA.

^2^Department of Biologic and Materials Sciences, University of Michigan, Ann Arbor, MI 48109, USA.

^3^Biointerfaces Institute, University of Michigan, Ann Arbor, MI 48109, USA.

^4^Department of Mechanical Engineering, University of Michigan, Ann Arbor, MI 48109, USA.

^5^Department of Biomedical Engineering, Tianjin University, Tianjin, P.R. China.

^6^Department of Cell and Developmental Biology, University of Michigan Medical School, Ann Arbor, MI 48109, USA.

^7^Section of Periodontics, University of California, Los Angeles School of Dentistry, Los Angeles, CA 90095, USA.

^§^These authors contribute equally.

*Correspondence to: [cxdeng@umich.edu](mailto:cxdeng@umich.edu) and [pkrebsbach@dentistry.ucla.edu](mailto:pkrebsbach@dentistry.ucla.edu)


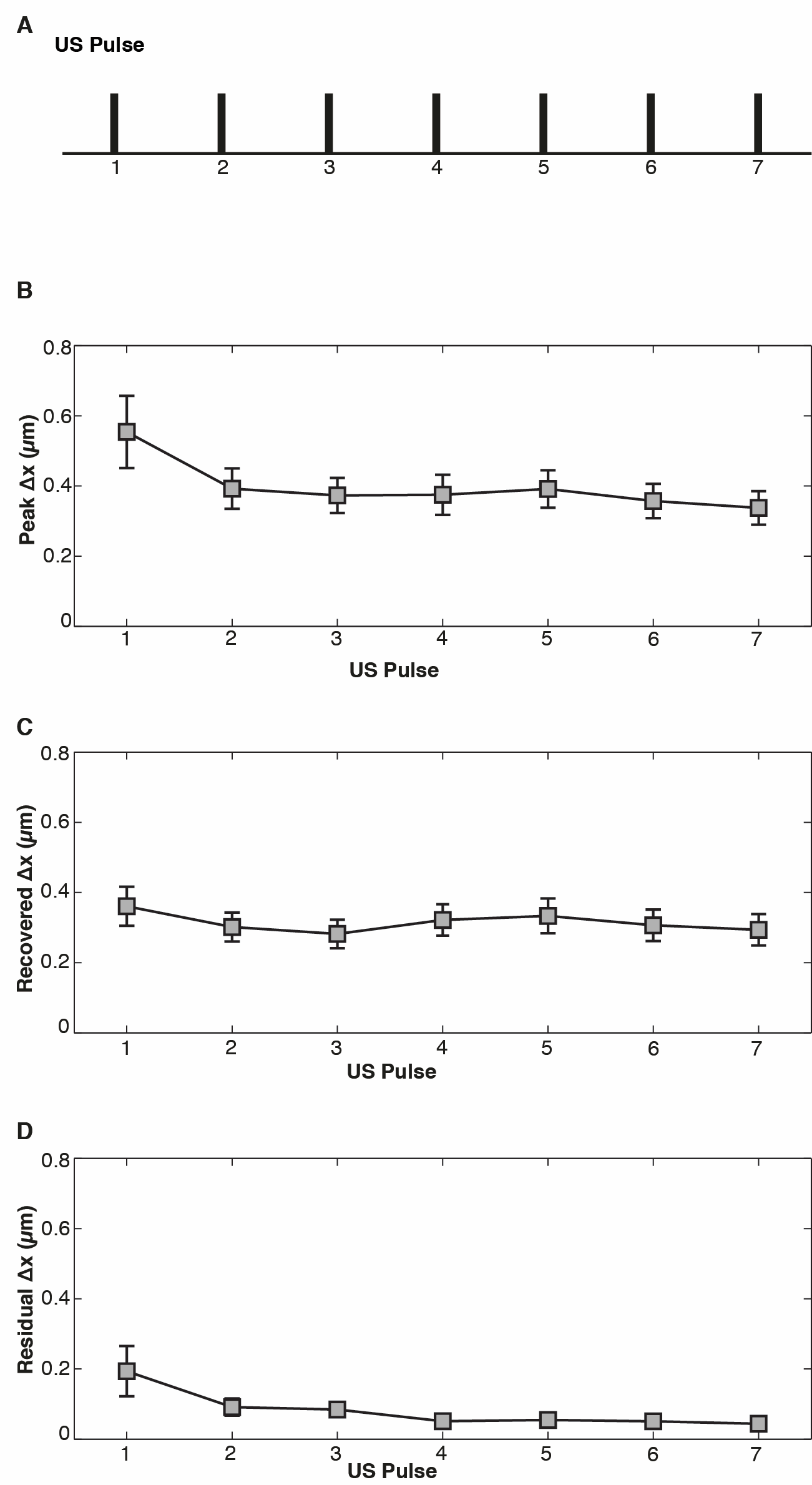


**Fig. S1**. MB displacements driven by pulsed US exhibit characteristic viscoelastic properties. **(A)** Schematic of US pulses with a pulse repetition frequency (PRF) of 1 Hz, duration of each pulse 50 ms, and acoustic pressure amplitude 0.03 MPa. **(B)** Peak displacements (Δx) of MBs during the first 7 US pulses (Mean ± SEM, *n* = 118). **(C)** Values of displacement recovery after each US pulse for the first 7 pulses (Mean ± SEM, *n* = 118). **(D)** Residual displacements of MBs after each US pulses for the first 7 pulses (Mean ± SEM, *n* = 118).

**Fig. S2**. ATC application using pulsed US excitation of integrin-bound MBs increased cellular contractile forces in single hESCs. **(A)** Bright field images and subcellular contractile force distributions in single hESCs at t = 0, 20 and 30 min in control group (-MB-US; top) and ATC treated group (+MB+US; bottom). ATC was applied at t = 0 for 30 min. Scale bars 10 µm. **(B)** Normalized contractile force of single hESCs cells in control group (-MB-US), ATC-treated group (+MB+US), and (+MB+US + Y27632 group (Y27) (10 µM) treated groups (Mean ± SEM, n = 8). Contractile force was normalized to pre-ATC treatment values.


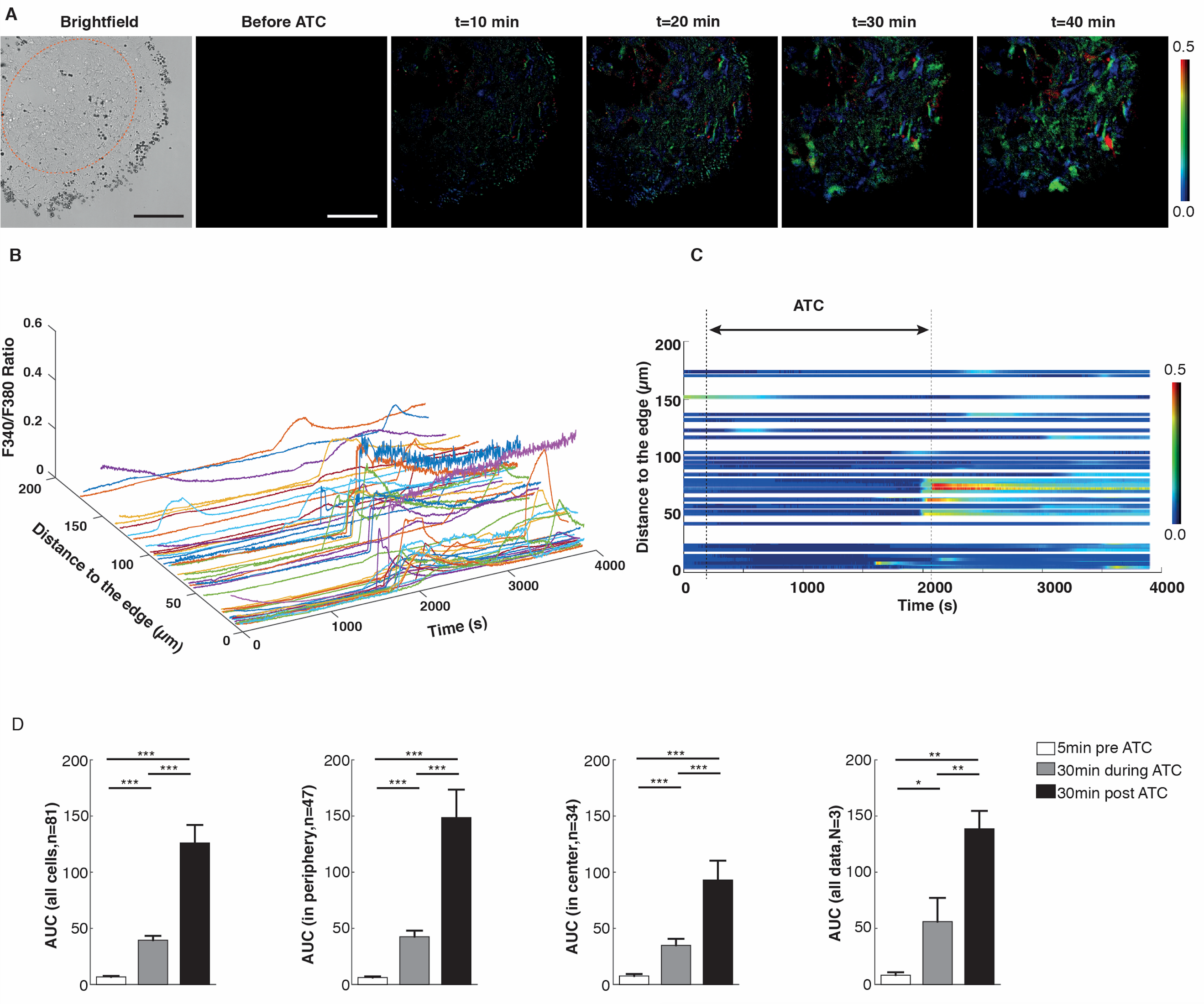


**Fig. S3**. ATC application generated calcium transients in ESC colony. **(A)** Bright field image showing microbubble preferentially attached to cells near the periphery with the number of bubbles in the central zone (within 70% of colony radius) and periphery zone (outside central zone) being 35 and 410 respectively; pseudo-color ratiometric calcium images (background subtracted) at t = - 5 min, 10 min, 20 min, 30 min, and 40 min. ATC was applied at t = 0 min for 30 min. Scale bars 100 µm. **(B)** Time-course of F340/380 ratio in cells in (A) (49 cells out of 190 cells). The ratio of fluorescence intensity excited at 340 nm and 380 nm (F340/380 Ratio) is linearly proportional to the intracellular calcium concentration. The y axis is the distance between a cell and the edge of the colony. Scale bars 100 µm. **(C)** 2D plot of F340/F380 in (B). **(D)** Area under curve (AUC) of F340/380 before, during, and after ATC application, *n* = 81 cells from *N* = 3 experiments. There were 34 cells in the central zone (70% of radius of colony) and 47 cells in periphery zone (region outside central zone) that exhibited calcium activities. *t* test p values < 0.05 (*), < 0.01(**), and < 0.001 (***).


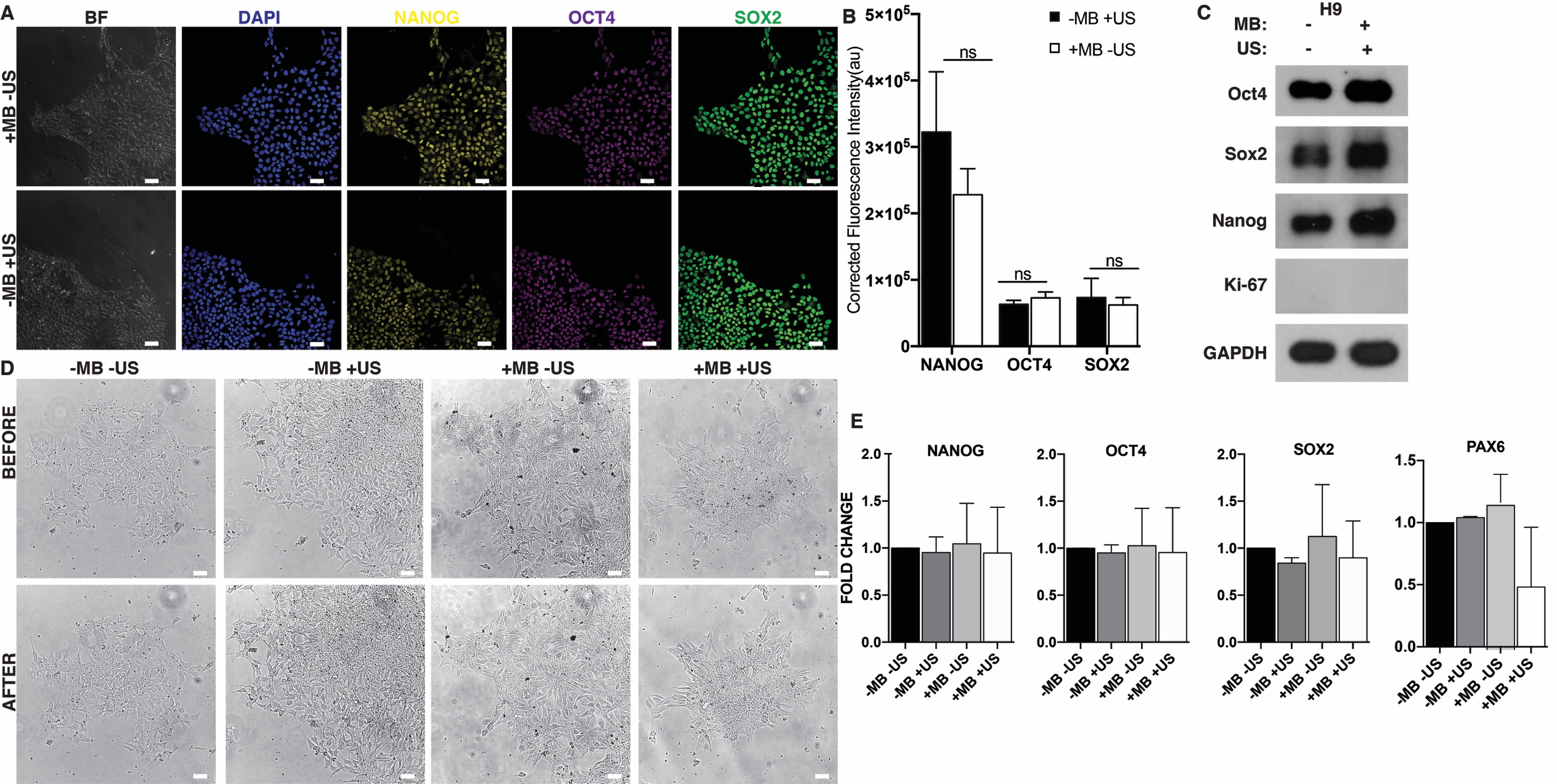


**Fig. S4.** A-B) Confocal micrographs of colonies of hESCs in control groups (+MB-US, -MB+US) exhibit no changes in expression of transcription factors. Scale bars 50 µm. C) Immunoblot analysis of control groups (+MB-US, -MB+US) exhibit no changes in expression of transcription factors. D) Brightfield images of hESC colonies before and after ATC treatmenr E) qRT-PCR analysis of Nanog, Oct4, Sox2, and Pax6.


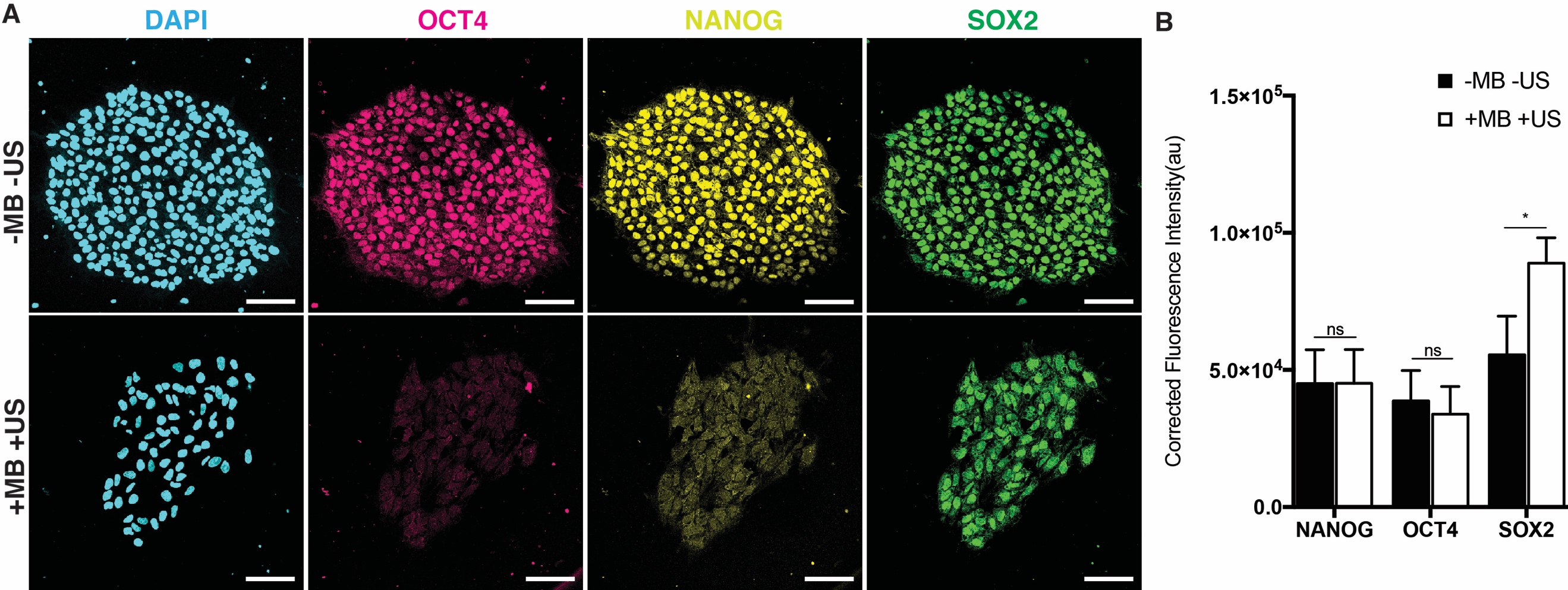


**Fig. S5**. ATC-induced decreases of nuclear Oct4 and Nanog in hESCs persisted for 48 hours. (A) Confocal fluorescence images of hESC colony stained with DAPI (blue), Oct4 (magenta), Nanog (yellow), and Sox2 (green) with and without ATC application after 48 hours culture. (B) Percentages of ATC applied hESCs with nuclear Oct4, Nanog, and Sox2 after 48 hours culture. Scale bars 50 µm. Quantifications were from 3 independent experiments with two replicates per experiment. Quantifications were mean ± SEM; unpaired t test p values < 0.001 (***).


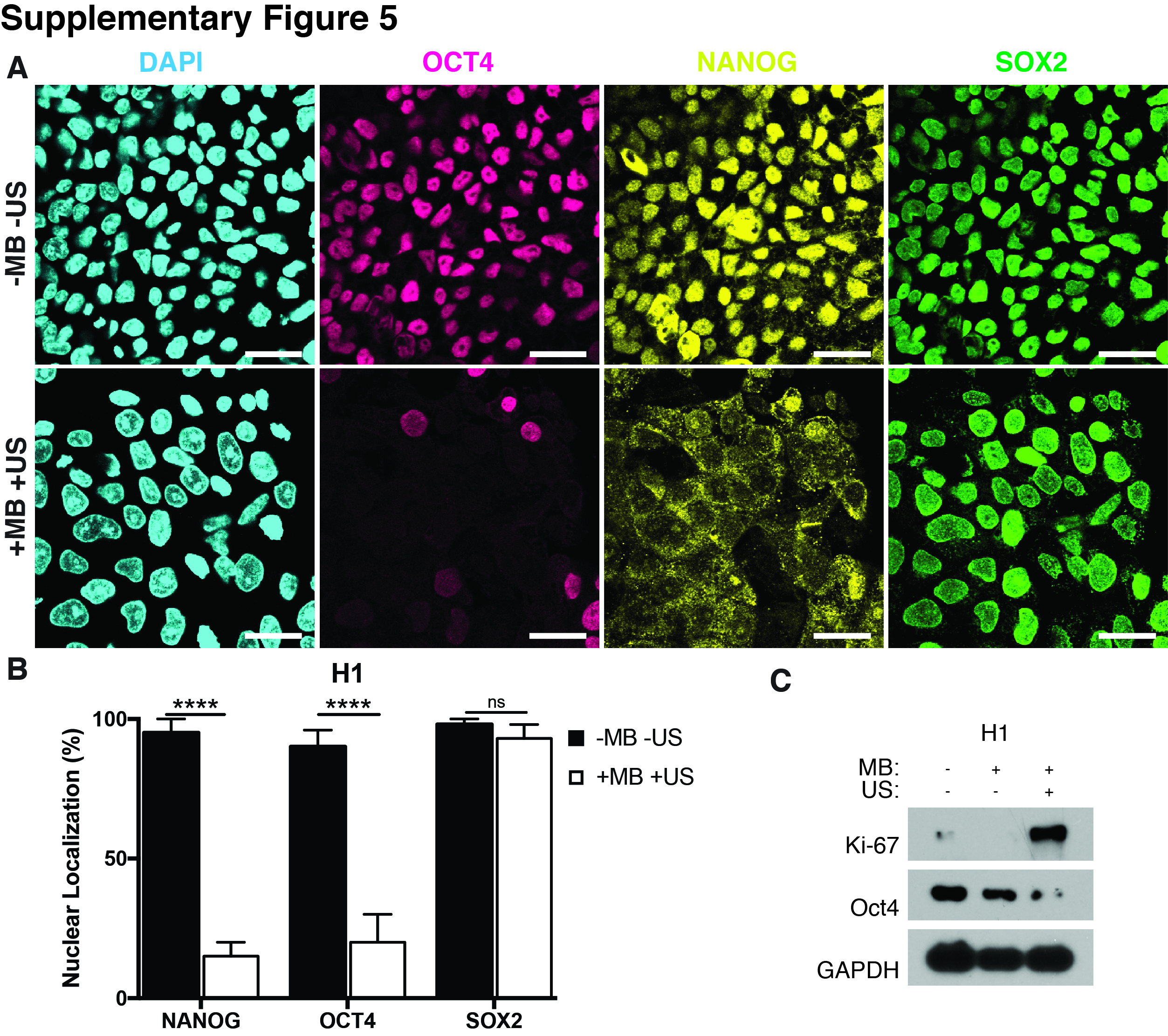


**Fig. S6.** ATC-induced changes in another hESC line H1. (A) Confocal fluorescence images of hESCs stained with DAPI (blue), Oct4 (magenta), Nanog (yellow), and Sox2 (green) with and without ATC application (30 min). (B) Percentages of hESCs with nuclear Nanog, Oct4, and Sox2 after ATC treatment compared with control. (C) Immunoblot analysis of protein expression in hESCs. Scale bars: 50 µm. All quantifications were from at least 3 independent experiments with two replicates per experiment. Unpaired t test p values < 0.05 (*), < 0.01(**), and < 0.001 (***); n.s, not significant.
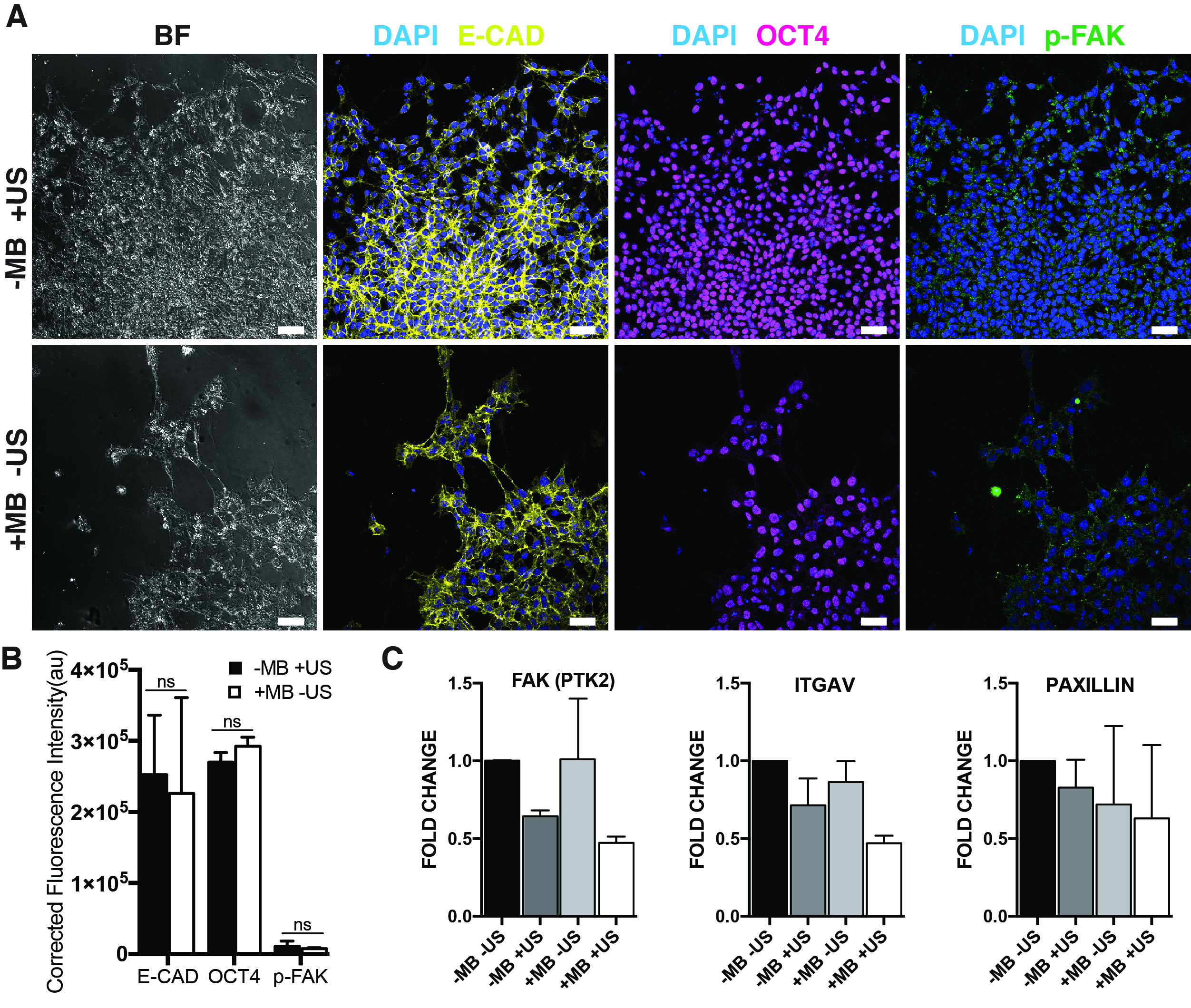


**Fig. S7.** A, b) Colonies of hESCs in control groups (+MB-US, -MB+US) exhibit no changes in expression of transcription factors. C) qRT-PCR analysis of FAK, ITGAV, and Paxillin. Scale bars 50 µm.


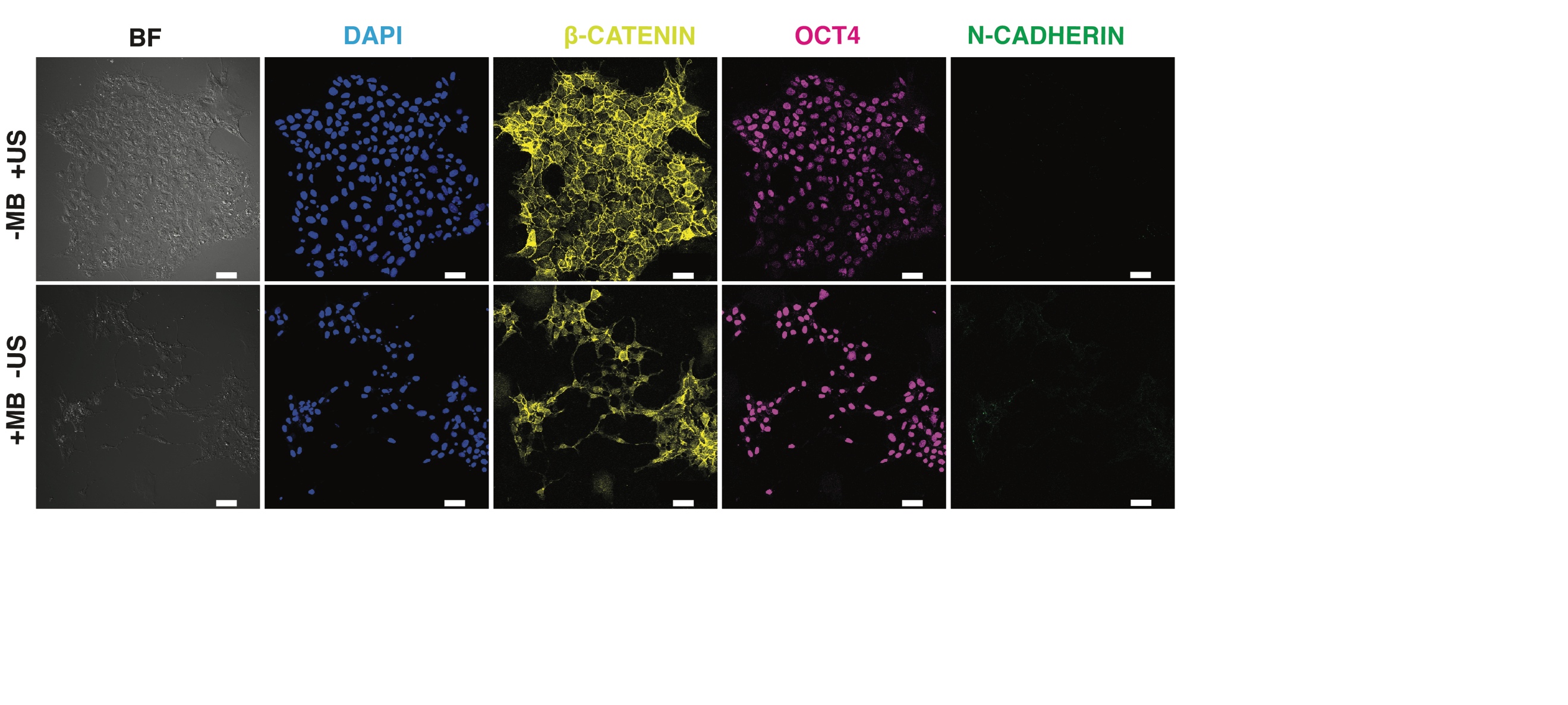


**Fig. S8:** Colonies of hESCs in control groups (+MB-US, -MB+US) exhibit no changes in expression of β-catenin, Oct4 and N-cadherin. Scale bars 50 µm.


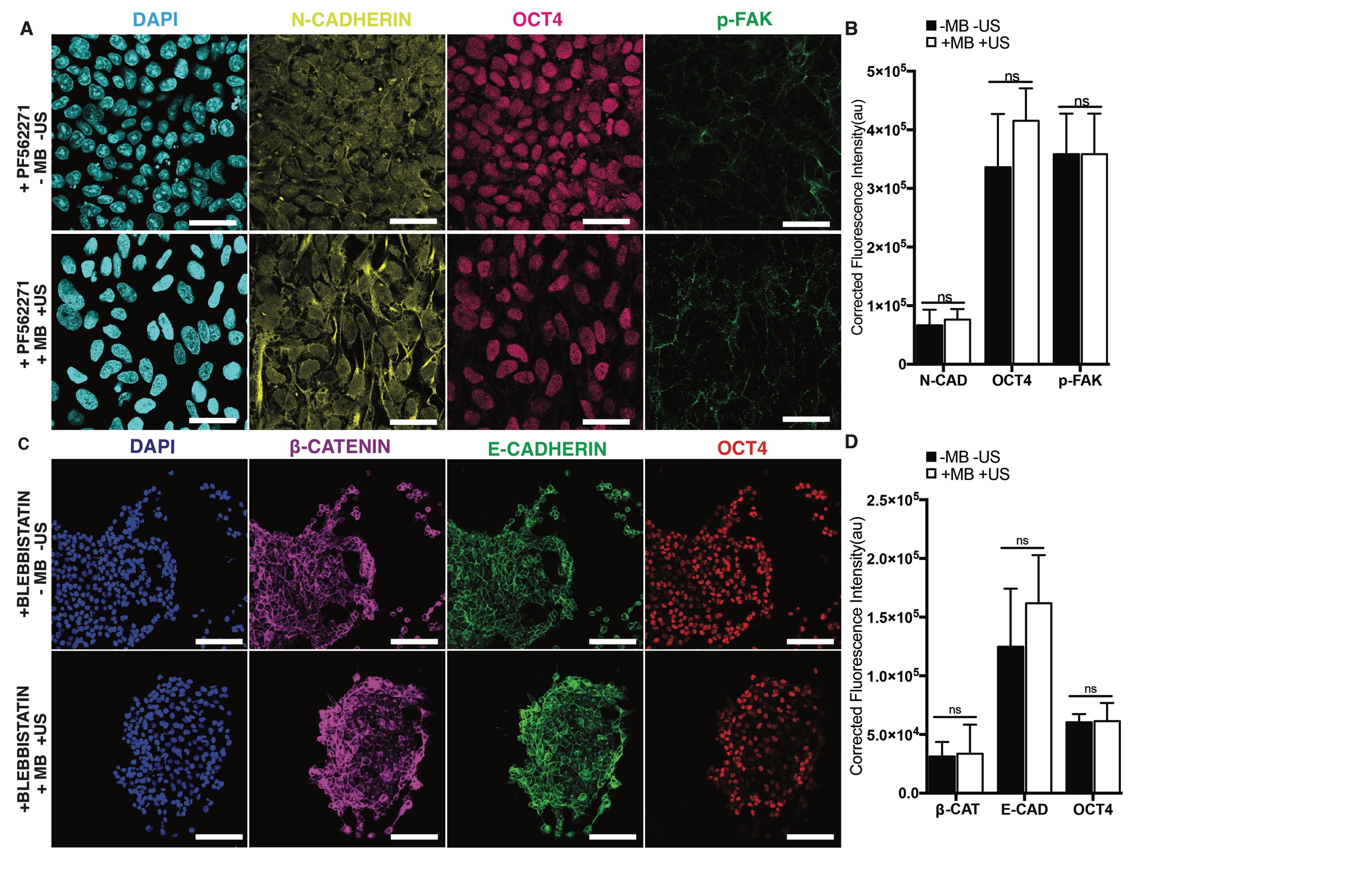


**Fig S9:** FAK inhibition abolished ATC-induced changes in hESCs. (A) hESCs treated with FAK inhibitor PF562271 showing DAPI (blue), N-cadherin (yellow), Oct4 (magenta), and pFAK (green) stain with and without ATC application. (B) Fluorescent intensity of N-cadherin expression and percentages of hESCs with Oct4 and pFAK expression in hESCs treated by PF562271 with and without ATC stimulation. (C) hESCs treated with blebbistatin stained with DAPI (blue), β-Catenin (pink), E-Cadherin (yellow), and Oct4 (red) with and without 30 min application of ATC. (D) Fluorescent intensities of β-catenin and E-cadherin expressions, and percentage of hESCs with Oct4 localization with and without ATC stimulation. Scale bars 50 µm. All quantifications were from at least 3 independent experiments with two replicates per experiment. Quantifications were mean ± SEM; unpaired t test p values < 0.05 (*), < 0.01(**), and < 0.001 (***); n.s. not significant.


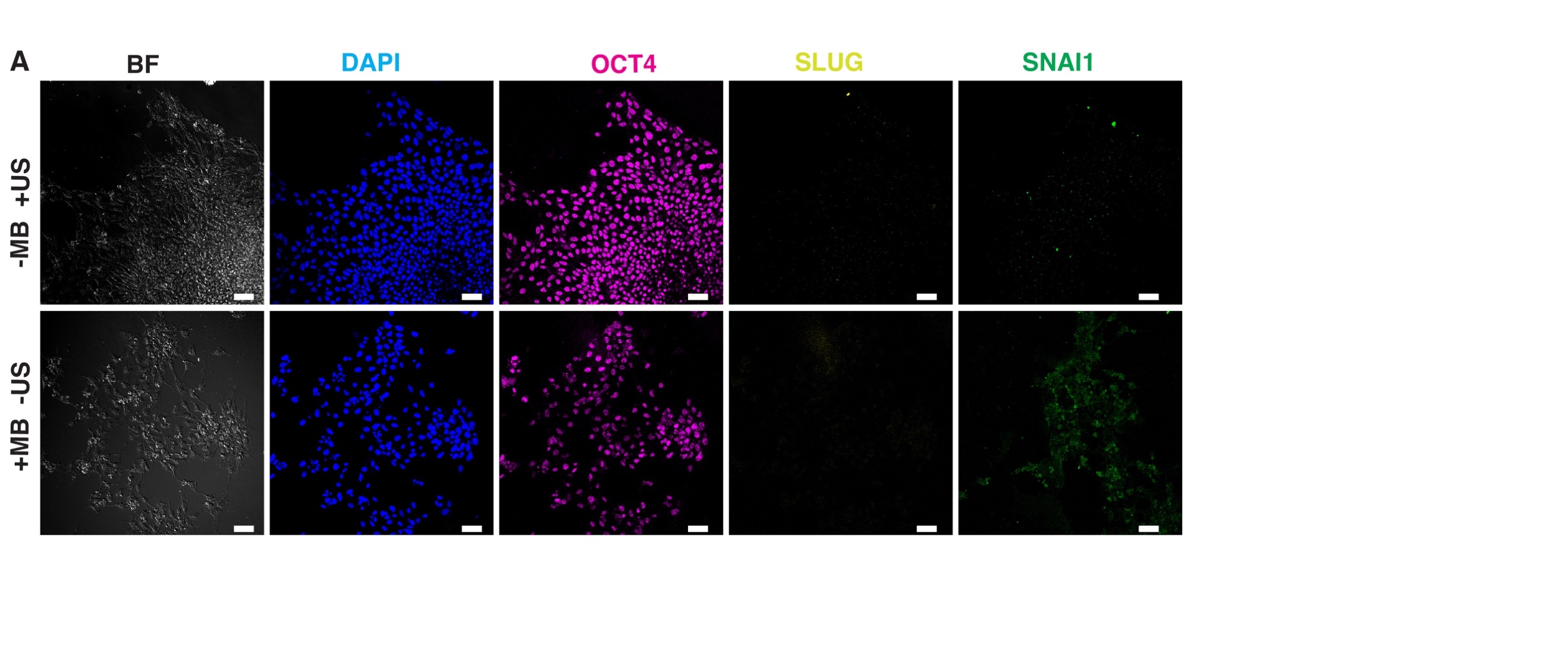


**Fig. S10:** Colonies of hESCs in control groups (+MB-US, -MB+US) exhibit no changes in expression of Slug and Snai1. Scale bars 50 µm.


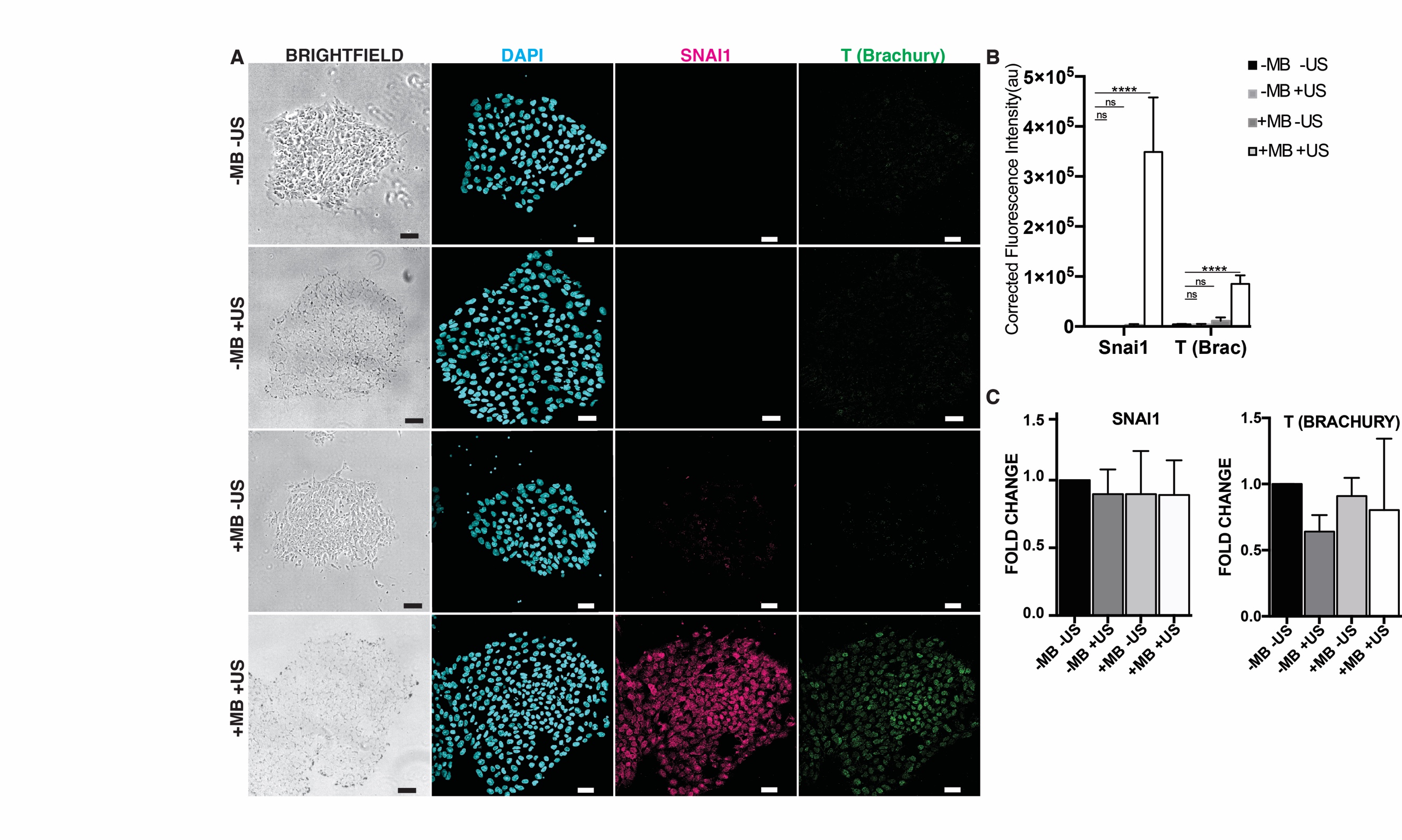


**Fig S11:** ATC application increased expression of Snail and T in hESC colonies. (A) hESCs were stained with DAPI (blue), Snail (purple), and T (Brachury) (yellow) with and without 30 min of ATC stimulation. Scale bars 50 µm. (B) Quantification of Snai1 and T expression after 30 min of ATC application compared with control group. Data from 3 independent experiments with two replicates per experiment. Unpaired t test p values < 0.05 (*), < 0.01(**), and < 0.001 (***). n.s. not significant.


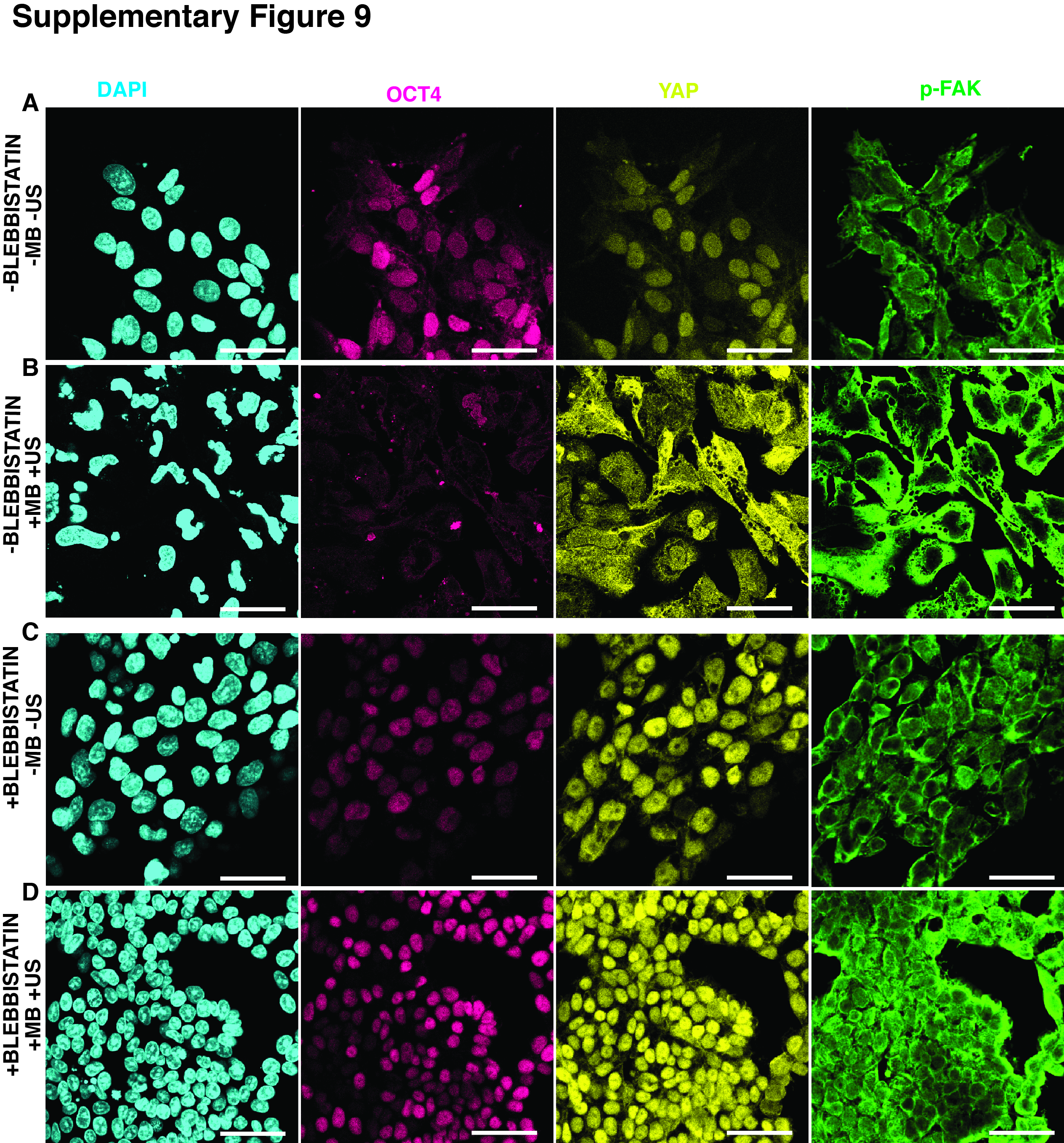


**Fig S12:** ATC-induced hESC differentiation required activation of FAK and NMII activity. hESCs with (A and B) and without (C and D) Blebbistatin treatment were stained with DAPI (blue), Oct4 (magenta), pFAK (yellow), and YAP (green), with and without 30 min of ATC stimulation. Scale bars 50 µm.


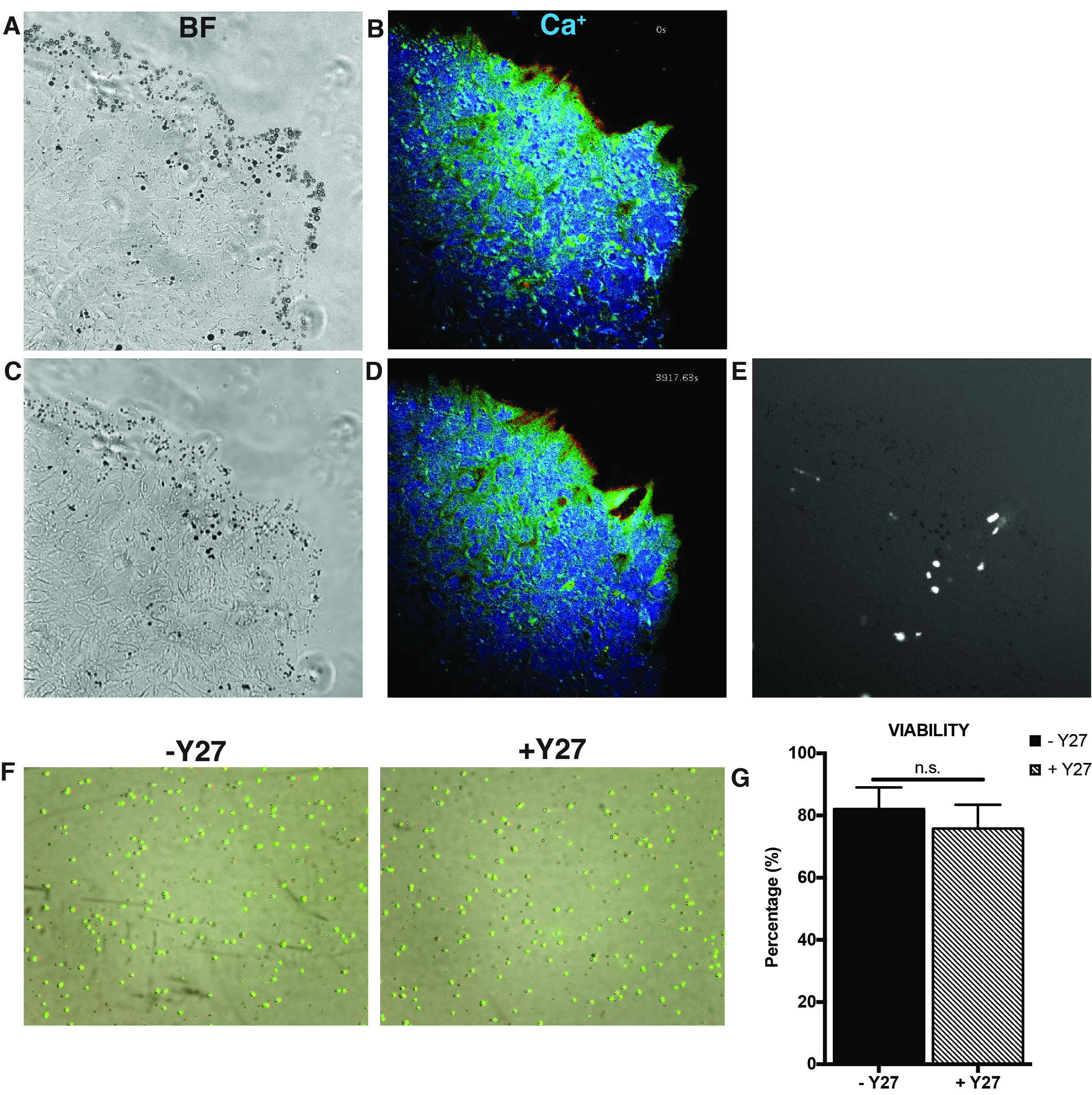


**Fig S13:** ATC-induced hESC differentiation does not lead cell death. Bright field images and pseudo color ratio matric calcium image (A, B) before ATC, and C,D) post entire fluorescent recording (5mins pre ATC, 30mins ATC and 30 mins post ATC). (E) PI image after the entire fluorescent recording. PI was added after the entire fluorescent recording. Cells treated with and without rock inhibitor (y27) to check viability using Acridine Orange/Propidium Iodide, green (live) cells (AO^+^), red (dead) cells (PI^+^) (F and G).


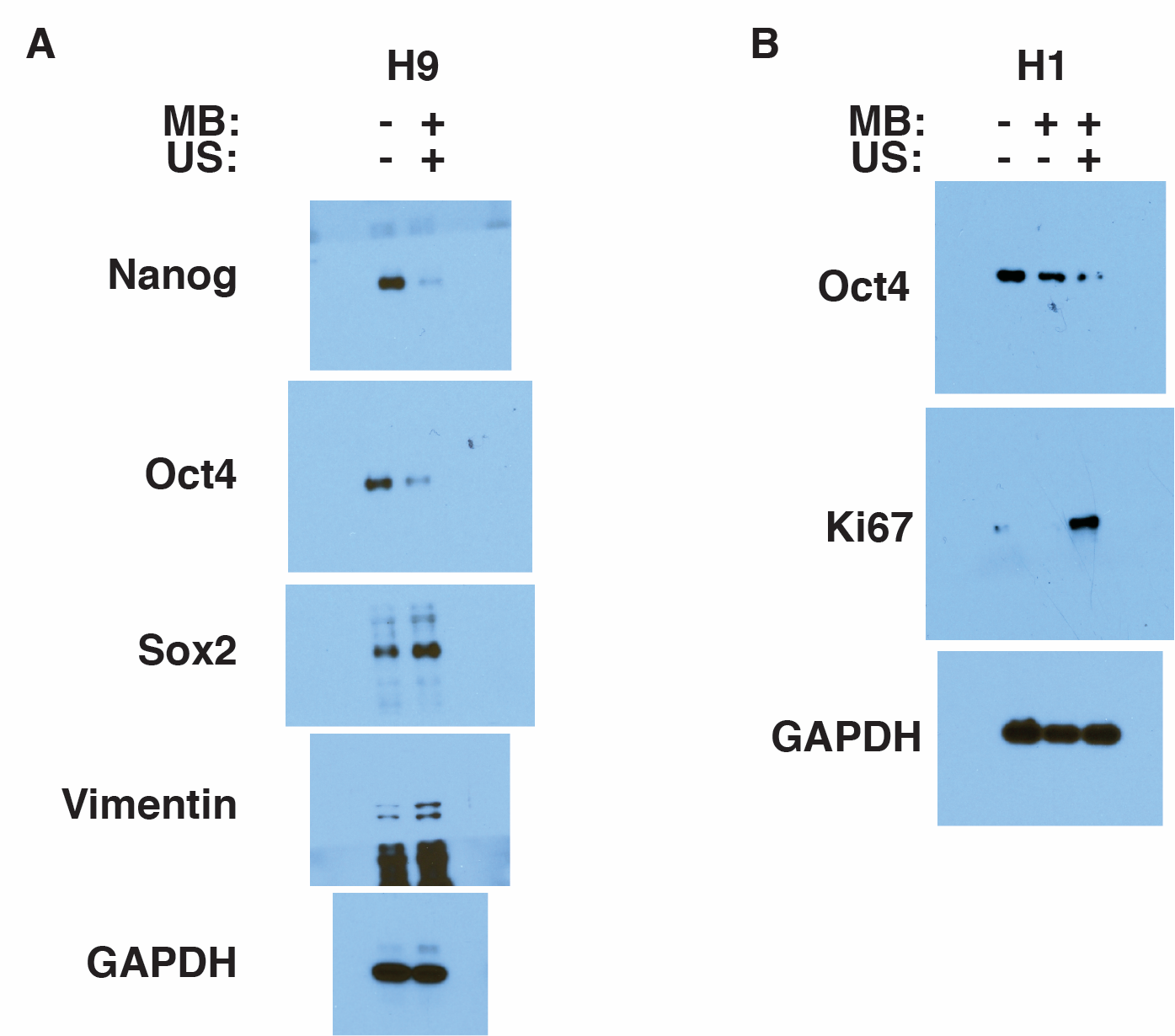


**Fig S14:** Original immunoblots films.
